# AZFa Y gene, *DDX3Y,* evolved novel testis transcript variants in primates with proximal 3´UTR polyadenylation for germ cell specific translation

**DOI:** 10.1038/s41598-022-12474-0

**Published:** 2022-05-27

**Authors:** P. H. Vogt, M-A. Rauschendorf, J. Zimmer, C. Drummer, R. Behr

**Affiliations:** 1 Division of Reproduction Genetics, Department of Gynecological Endocrinology and Fertility Disorders, University Women Hospital, Im Neuenheimer Feld 440, D-69120 Heidelberg, Germany; 2 Molecular Health GmbH, Kurfürsten-Anlage 21, D-69115 Heidelberg, Germany; 3 Platform Degenerative Diseases, German Primate Center, Leibniz Institute for Primate Research, Kellnerweg 4, D-37077 Göttingen, Germany

**Keywords:** Developmental biology, Evolution, Genetics, Molecular biology

## Abstract

Translational control is a major level of gene expression regulation in the male germ line. *DDX3Y* located in the AZFa region of the human Y chromosome encodes a conserved RNA helicase important for translational control at the G1-S phase of the cell cycle. In human, DDX3Y protein is expressed only in premeiotic male germ cells. In primates, *DDX3Y* evolved a second promoter producing novel testis-specific transcripts. Here, we show primate species-specific use of *a*lternative *p*oly*a*denylation (APA) sites for these testis-specific *DDX3Y* transcript variants. They have evolved subsequently in the 3´UTRs of the primates´ *DDX3Y* transcripts. Whereas a distal APA site (PAS4) is still used for polyadenylation of most *DDX3Y* testis transcripts in *Callithrix jacchus*; two proximal APAs (PAS1; PAS2) are used predominantly in *Macaca mulatta*, in *Pan trogloydates* and in human. This shift corresponds with a significant increase of DDX3Y protein expression in the macaque testis tissue. In chimpanzee and human, shift to predominant use of the most proximal APA site (PAS1) is associated with translation of these *DDX3Y* transcripts in only premeiotic male germ cells. We therefore assume evolution of a positive selection process for functional *DDX3Y* testis transcripts in these primates which increase their stability and translation efficiency to promote its cell cycle balancing function in the human male germ line.

## Introduction

The human AZoospermia Factor a (AZFa) Y gene, *DDX3Y* (*DEAD-Box Helicase 3, Y-linked*; OMIM # 400,010), is located in the proximal part of the long arm of the Y chromosome (Yq11.1)^1,2^. Its deletion on the Y chromosome of infertile men, together with *USP9Y* (*Ubiquitin-Specific-Protease 9, Y-linked*)*,* designated as “complete AZFa deletion” causes a severe testicular pathology, the Sertoli Cell Only (SCO) syndrome^3^. *USP9Y* deletions can be inherited by fertile men^4,5^ and its expression in germ cells was found only in late spermatids^6^. *DDX3Y* transcripts are translated only in premeiotic male germ cells^7^. It has been therefore assumed that the SCO syndrome associated with complete AZFa deletions occurs only if this deletion also comprises *DDX3Y*^8,9^. Consequently, the *DDX3Y* gene has been designated as major AZFa gene^10^.

AZFa is part of the Azoospermia Factor (AZF) locus mapped cytogenetically 45 years ago to Yq11, the euchromatic part of the long arm of the human Y chromosome^11^. Molecular deletion mapping of AZF on the Y chromosome of large numbers of infertile men with normal karyotype, 46,XY, revealed that the functional Y gene content of AZF can be split and mapped to three distinct molecular Yq11 subintervals, coined: AZFa, AZFb, and AZFc, respectively. AZFa deletion causes complete absence of germ cells in the patients testis tubules, AZFb deletion causes meiotic arrest, AZFc deletion causes severe hypospermatogenesis^3,12^.

*DDX3Y* has a functional homologue on the short arm of the X chromosome (Xp11.4), *DDX3X* (*DEAD-Box Helicase 3, X-linked*; OMIM #300,160)^13^. Both belong to the strongly conserved PL10 subfamily of DEAD box RNA helicases^14^. They are functionally involved in the control of transcripts translation initiation in the cytoplasm, respectively, in the control of cell cycle progression at the G1-S phase^15–17^. Accordingly, *DDX3Y* expression can rescue the cell cycle function of *DDX3X* after mutation in hamster cells^18^ and is involved in the cell cycle control of early spermatogonia^19^.

The *DDX3Y* male germ cell function may have evolved first 80 million years ago (mya) after separation of the primate lineage from the rodent lineage. The mouse homologue, *Ddx3y*, although located with *Usp9y* in a similar syntenic block together on the mouse Y chromosome^20^ is not functional for mouse male germ cell development^21^. In primates, a function of *DDX3Y* in the male germ line was first indicated by analyzes of some novel *DDX3Y* transcript variants with longer 5´UTR extensions found only in the testis tissue of old world (catarrhini) and new world (platyrrhini) monkeys^22^. These were generated from a novel and germ cell specific distal *DDX3Y* promoter domain most likely activated by amplification of a ~ 100 bp long genomic sequence block. It was coined “*MSY2 minisatellite*” and is located ~ 1 kb upstream of the primates *DDX3Y* gene coding region^23^. *MSY2* has 2 copies on the Y chromosome of non-human primates and amplified those in a second step only recently, eventually presenting 3 or 4 copies on the human Y chromosome^22,23^. Comparative mapping of 5´UTR start sites of the *DDX3Y* testis specific transcripts on the Y chromosome of distinct primates revealed transcripts starting in *MSY2* first in old world monkeys^22^. Interestingly, these germ cell specific *DDX3Y* transcripts have shortened their 3´untranslated sequence region (3´UTR) lengths significantly; all are polyadenylated in their proximal 3´UTR^22^.

All *DDX3Y* transcripts in human somatic cells and tissues without DDX3Y protein expression contain ~ 2.4 kb long 3´UTRs^7,13^. Hence 3´UTR shortening might be an essential prerequisite for *DDX3Y* translation in male germ cells. We therefore assume that *DDX3Y* transcript variants that are translated have developed short 3´UTRs during primate evolution.

Indeed, shortening of 3´UTRs of gene transcripts by the use of proximal *a*lternative *p*oly*a*denylation sites (APAs) is a general translation control mechanism for ubiquitously transcribed genes to achieve tissue specific protein expression^24,25^. The use of APAs has been shown especially for germ cell transcripts whose translation is restricted to only a specific phase of spermatogenesis^26^.

Neither the pattern of functional APAs in the 3´UTR of *DDX3Y* germ line transcripts in primates is known, nor anything about their translation capacities in these species. In this paper, we therefore present a comparative functional analyzis of *DDX3Y* 3´UTR transcript variants in kidney, liver and testis tissue of a new world monkey, the common marmorset (*Callithrix jacchus*), an old world monkey, the rhesus macaque (*Macaca mulatta*), and a hominoid, the chimpanzee (*Pan troglodytes*), and compare the APA usage patterns with that found in the corresponding human tissues. Comparative analyzis of DDX3Y protein expression by Western blotting in the same primate tissues was used to reveal the evolutionarily time point of their translational restriction to the male germ line. By immuno-histochemical analyzes of testicular tissue sections from these primate species with a DDX3Y specific antibody we analyzed, whether the putative functional requirement for the DDX3Y RNA helicase in primate male germ cells is similar or divergent from that found in the human male germ line.

## Results

### *DDX3Y* primate transcripts evolve novel APA site patterns in their 3´UTR sequence

The mouse *Ddx3y* gene 3´UTR sequence is reported with 2573 nucleotides (nts) in the data base (GenBank acc. No.: NM_012008.2). Two alternative polyadenylation (APA) sites with a canonical PAS consensus sequence (*“AATAAA”*) were reported by Vong and coworkers^27^. The transcripts are expressed in each mouse tissue (GenBank acc.: NM_012008). However, nothing is known about the pattern of APA sites in the 3´UTR of primate *DDX3Y* genes.

In this study, we analyse the pattern of putative APA sites in the corresponding genomic 3´UTR sequences of the *DDX3Y* genes on the Y chromosome of human and the *N*on-*H*uman *P*rimates (NHP), *Callithix jacchus, Macaca mulatta*, and *Pan trogloydates,* respectively.

**Figure 1:**
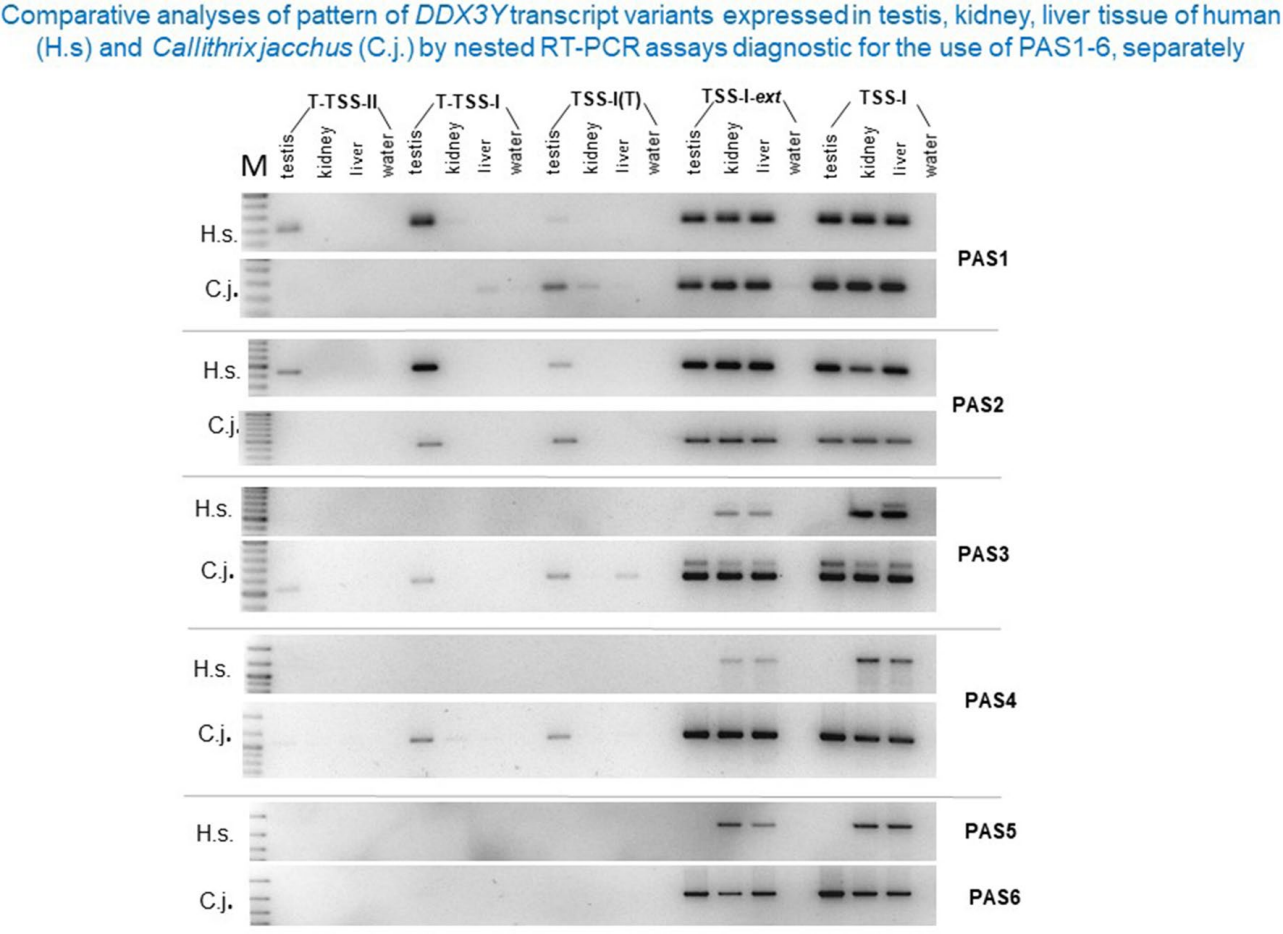
Comparative pattern of amplification products produced in the second round of the nested RT-PCR assays with the common forward primer spanning *DDX3Y* exon 16–17 (see also Supplementary Fig. 3S). They are diagnostic for presence or absence of the *DDX3Y* transcript variants expressed in testis, kidney and liver tissue of human (H.s.) and *Callithrix jacchus* (C.j.) as described in the Material & Methods section. Vertical lanes separate gel slot triplets (testis, kidney, liver) of the distinct transcriptional start sites (TSSs) known from Rauschendorf et al.^22^ to be “testis specific”: “T-TSS-II” and “T-TSS-I” in the distal *DDX3Y* promoter region, respectively, “TSS-I(T)” in the proximal *DDX3Y* promoter region. Additionally, two more slot triplets are displayed which show amplification products of *DDX3Y* transcripts, when starting at “TSS-I-*ext*”, respectively, at “TSS-I”. These transcriptional start sites reflect the variation of 5´UTR extensions found in human testis and somatic *DDX3Y* transcripts; its variability is typical for CpG island containing promoter domains^22^. The four horizontal lines separate the amplification products got with the “*inner reverse”* primer associated with PAS1, PAS2, PAS3, PAS4, and PAS5 as shown schematically in Fig. S3. In human, *DDX3Y* testis transcripts starting at T-TSS-I/II, respectively, at TSS-I(T) are mainly poyadenylated at PAS1 (“T-TSS-I/II”), respectively at PAS2 (“TSS-I(T)”); no amplification products became visible in the RT-PCR assays for presence of PAS3-5. In *Callithrix jacchus*, *DDX3Y* testis transcripts starting at T-TSS-I and TSS-I(T) are polyadenylated at PAS4 because amplification products are visible in the PAS1-4 RT-PCR assays, but not for PAS6 (note: PAS5 in human is identical to PAS6 in Callithrix; see supplementary information: Fig. 1S). Somatic kidney and liver *DDX3Y* transcripts are polyadenylated at PAS5 in human and at PAS6 in *Callithrix jacchus* because amplification products becomes only visible when the *“outer forward”* primers associated with “TSS-I-*ext*” and “TSS-I” have been used in the first round. For further discussion, see main text.

Comparative CLUSTAL multiple sequence alignments revealed absence of the mouse *Ddx3y* 3´UTR APA sites described by Vong et al.^27^. Further 7 APA sites, now mapped additionally “in silico” (using CLUSTAL see M&M) in the mouse *Ddx3y* 3´UTR, were also not present in any of the corresponding 3´UTRs of the human and NHP *DDX3Y* genes (supplementary information: Fig. 1S). Only PAS11, the last canonical (*“AATAAA”*) PAS motif in the long mouse *Ddx3y* 3´UTR sequence (NM_012008.2), was conserved in the human and NHP long *DDX3Y* 3´UTR sequence and coined “PAS5”, respectively, “PAS6” in *Callithrix jacchus* (supplementary information: Fig. 1S). In human, *DDX3Y* transcripts with the long 3´UTR are found in each tissue analyzed^22^.

However, we found novel APA sites along the aligned 3´UTR sequences of the primate *DDX3Y* genes especially in the proximal 3´UTR. A canonical PAS motif (“*AATAAA*”) was found 175 nucleotides (nts) upstream of the translation stop codon in the new and old world monkey species (“PAS1”). In chimpanzee and human, PAS1 has evolved 60 nts more upstream in their 3´UTR sequences with sequence motif “*ATTAAA*”. It is the most common PAS variant with 70% 3´UTR cleavage activity^28^. More novel APA sites (“PAS2” and “PAS3”) were found 163 nts, respectively, 351 nts more downstream in the proximal 3´UTRs. They are conserved in all primate *DDX3Y* gene transcripts including human; PAS2 is a canonical “*AATAAA*” motif, PAS3 the “*ATTAAA*” variant **(**supplementary information: Fig. 1S). This stepwise evolution of novel APA sites in the primate *DDX3Y* proximal 3´UTRs suggests selection for increased cellular stability and translation efficiency of the *DDX3Y* transcripts in primates^24–26^.

More novel APA sites were also found in the distal part of the primate *DDX3Y* transcripts 3´UTR (“PAS4” in all primates; “PAS5” only in *Callthrix jacchus* (supplementary information: Fig. 1S). The most distal 3´UTR PAS site in human and NHP is associated with the longest *DDX3Y* transcripts^22^ and corresponds to PAS11 in the mouse *Ddx3y* 3´UTR. It represents “PAS5” in human, chimpanzee and rhesus and “PAS6” in *Callithrix jacchus*, respectively (Supplementary information: Fig. 1S).

### Polyadenylation process of *DDX3Y* testis transcripts in primates shifts to proximal 3´UTR in old world monkeys

**Figure 2:**
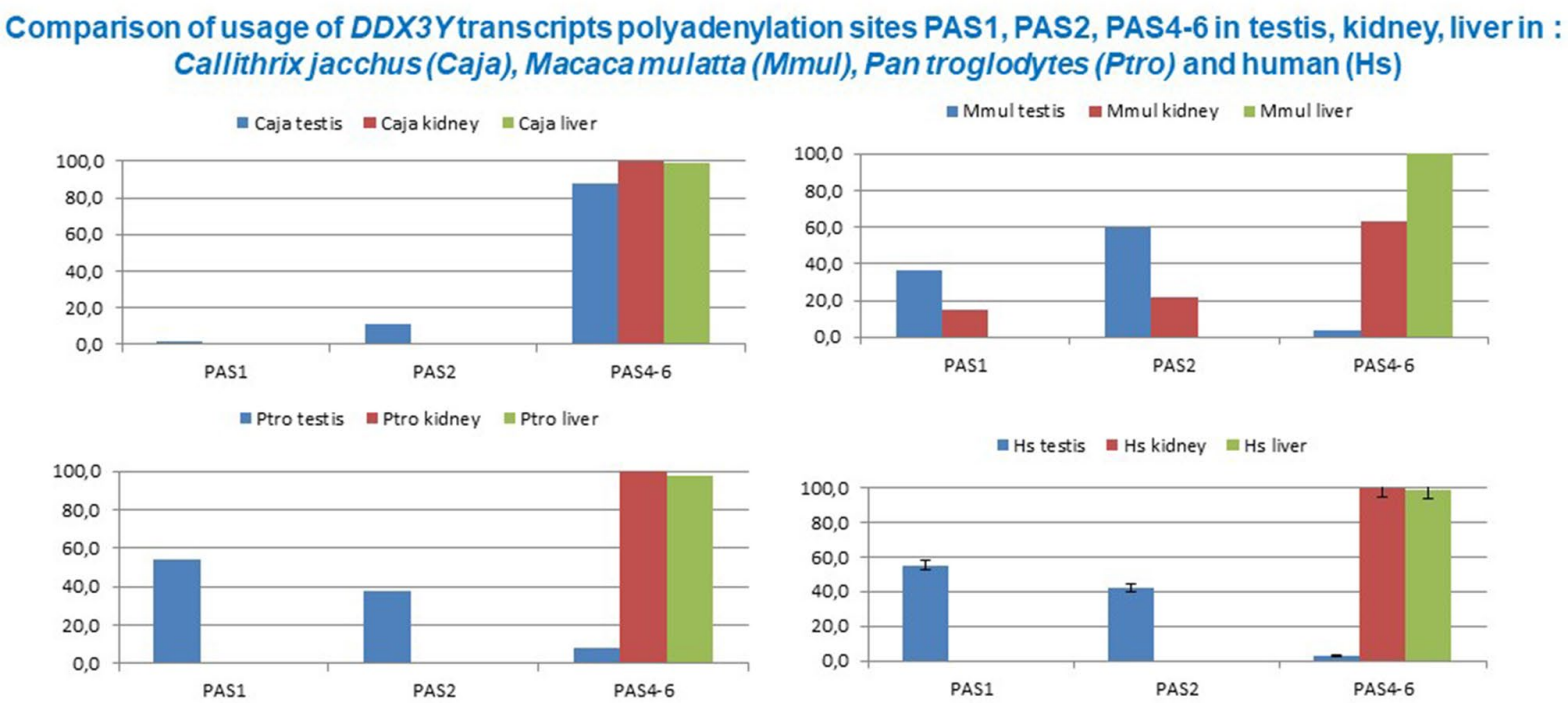
Quantitative evaluation of polyadenylation processing of *DDX3Y* transcripts in testis, kidney, and liver tissue when using one of the proximal 3´UTR polyadenylation sites, (PAS1, PAS2), respectively, one of the distal PAS4-6 in *Callithrix jacchus, Macaca mulatta*, *Pan troglodytes* and human. Comparison of *DDX3Y* transcripts was performed in parallel with three distinct TaqMan probes (“PAS1”; “PAS2”, “PAS4-6”) by appropriate TaqMan assays in as described in Material & Methods section (for specification and location of primer sequences see supplementary information: Table S3). Most *DDX3Y* transcripts in *Callthrix jacchus* (Caja) are processed for polyadenylation in the distal 3´UTR part as assessed by TaqMan probe “PAS4-6”. Only in testis tissue about 10% of the *DDX3Y* transcripts become polyadenylated after PAS2 and less than 1% at PAS1. This corresponds to the data of our RT-PCR assays shown in Fig. 1. In *Macaca mulatta* (Mmul), more than 90% of the *DDX3Y* testis transcripts and more than 30% of *DDX3Y* transcripts in kidney are polyadenylated at PAS1 and PAS2, respectively. In liver tissue, *DDX3Y* transcripts are processed for polyadenylation only in the distal 3´UTR containing PAS4-6. In *Pan troglodytes* (Ptro) and human (Hs), all *DDX3Y* transcripts expressed in kidney and liver are processed in distal 3´UTR after PAS4-6, whereas in testis tissue PAS1 in the proximal 3´UTR is the major polyadenylation site. Less than 5% of *DDX3Y* transcripts in the testis tissue are processed for polyadenylation after PAS4-6. Numbers on the scale at the left of each diagram represent percentage values.

Nothing is known yet about use of the novel APA sites for polyadenylation of primate *DDX3Y* transcripts. It is also unknown, whether the testis specific *DDX3Y* transcripts starting from the testis specific promoter domains of the primate *DDX3Y* genes (Supplementary informations; Fig. 2S) predominantly use one of the proximal 3´UTR APA sites (PAS1, PAS2) like shown in human^7,22^.

To answer this question experimentally, we developed a sensitive nested RT-PCR protocol for amplification of any known *DDX3Y* transcript variant expressed in human and NHP testis, kidney and liver tissue, separately. For this purpose, we used a series of forward and reverse primer sets. In the first round, the primers span the transcript sequences from their 5´UTR start sites (*forward* primer set) to their 3´UTR polyadenylation sites (*reverse* primer set) (Supplementary informations: Fig. 2S; Table S1). These RT-PCR assays produce thus only amplification products when the *DDX3Y* transcript variant selected by a specific *forward* and *reverse* primer pair will span the complete transcript sequence from exon 1 to exon 17, downstream the used 3´UTR PAS. Transcript variants polyadenylated at PAS1 will thus only be amplified with the *reserve* primer upstream of PAS1, but not with those upstream of PAS2-5, because they are located downstream of PAS1; transcripts polyadenylated at PAS5 will be amplified also with the *reverse* primers upstream of PAS1-4 because all are located upstream of PAS5.

To visualize each amplification product of this first round, a second round of RT-PCR experiments was required (“nested RT-PCR assay”). It uses a common *forward* primer spanning exon 16–17 which is present in each amplification product of the first round. This site will be combined in the second round with one of the so called *“inner primers reverse*” associated with each PAS in the 3´UTR because located upstream of the *“outer primer”* set (Supplementary information: Fig. S3). In this way, amplification products in the second round become diagnostic for the use of a specific polyadenylation site by one of the distinct *DDX3Y* transcript variants (Supplementary information: Table S2; for further details see Material & Methods section).

Main finding was a shift of the predominantly used polyadenylation site for *DDX3Y* testis transcripts from PAS4 in the new world monkey, to PAS2 in the old world monkey when starting in the testis specific distal promoter domain at the T-TSS-I/ T-TSS-II site, respectively, in the proximal promoter at TSS-I(T) (Fig. 1). In human, the proximal TSS-I(T) start in exon1 was also only found in *DDX3Y* testis transcripts^22^. Interestingly, although PAS4 is still conserved in the *DDX3Y* 3´UTR of *Macaca mulatta* (Supplementary information: Fig. 1S) testis transcripts have shifted their mainly used polyadenylation signal to PAS2 in this old world monkey (Supplementary information: Table S2). In the human and Pan 3´UTR sequence, novel mutations have then destroyed the PAS4 motif (Supplementary informations: Fig. 1S). Somatic *DDX3Y* transcripts are mainly starting in the proximal *DDX3Y* promoter domain at TSS-I in all primates including human. They become polyadenylated at PAS5, which corresponds to PAS6 in the 3´UTR of *Callithrix jacchus* (Fig. 1).

The amount of *DDX3Y* testis transcripts starting in the proximal promoter domain around TSS-I(T) and polyadenylated at PAS2 is reduced in human and Pan when compared with that found in Macaca and Callithrix (supplementary information: Table S2). Instead of this, the distal testis specific promoter domain seems to become more activated in both species (shown for human in: Fig. 1). The testis specific transcription of *DDX3Y* from its distal promoter domain, all polyadenylated in proximal 3´UTR becomes thus first prominent after separation of the platyrrhini from the catarrhini and hominoid primate lineages, i.e. ~ 46 Mya (Fig. 1; supplementary information: Table S2).

### Polyadenylation of *DDX3Y* testis transcripts at PAS1 becomes first predominant in *Pan troglodytes* and human

In human, 3´RACE (*R*apid *A*mplification of *C*-DNA Ends) experiments had revealed that not only PAS2, but also PAS1 is used for polyadenylation of the *DDX3Y* testis transcripts when starting from their distal promoter domain^22^. Quantitative discrimination between the two options in the PAS1 and PAS2 nested RT-PCR assays (Fig. 1) is not possible because of similar intensities of the RT-PCR amplification products.

We, therefore, compared the amount of *DDX3Y* transcripts polyadenylated at PAS1 and at PAS2 by appropriate TaqMan assays also quantitatively in testis, kidney, and liver tissue of each NHP and human. The third TaqMan probe (“PAS4-6”) was designed to bind upstream of PAS4-6 to collectively determine the amount of *DDX3Y* transcripts polyadenylated in the distal 3 `UTR at PAS4, respectively, the more distally located PAS5/PAS6 sites (Fig. 2).

In *Callithrix jacchus,* most *DDX3Y* transcripts in each tissue are polyadenylated in the distal 3´UTR, i.e., downstream of the location of the “PAS4-6” TaqMan probe. This finding confirms thus the results of our nested RT-PCR experiments; testis specific *DDX3Y* transcripts are polyadenylated downstream of PAS4 and somatic *DDX3Y* transcripts downstream of PAS6, respectively, in this new world monkey (Fig. 1). Quantitative comparison additionally revealed that about 10% of the testis *DDX3Y* transcripts were processed for polyadenylation at PAS2. Even PAS1 in the proximal 3´UTR seems to be used although only for less than 1% of all testis transcripts (Fig. 2).

In *Macacca mulatta,* polyadenylation at PAS2 is predominantly used for the *DDX3Y* testis transcripts (65%)*,* whereas PAS1 is used only in ~ 35% of them. Interestingly, rhesus monkey kidney *DDX3Y* transcripts also use PAS1 in ~ 15% and PAS2 in ~ 20% for their 3´UTR polyadenylation process. In *Pan troglodytes* and human, only testis *DDX3Y* transcripts are polyadenylated in proximal 3´UTR. They predominantly use PAS1 with ~ 58% and PAS2 with ~ 40% in their testis transcripts (Fig. 2). A small fraction of the human and chimpanzee testicular *DDX3Y* transcripts (< 5%) becomes polyadenylated in their distal 3´UTR. In all NHP species analyzed, all somatic *DDX3Y* transcripts are processed for polyadenylation in their distal 3´UTR like in human (Fig. 2).

These data extend our nested RT-PCR results and confirm that in primate testis tissue the process of polyadenylation along the 3´UTR of the *DDX3Y* gene transcripts has shifted also quantitatively during primate evolution towards the predominant use of PAS2 in *Macaca mulatta* and of PAS1 in the hominoid *Pan troglodytes* being comparable to human.

### DDX3Y protein expression evolved gradually towards testis specificity in hominoids

In order to analyze, whether the observed shift of the polyadenylation process of *DDX3Y* testis transcripts to the proximal 3´UTR is accompanied by (i) an increase in their translation efficiency and (ii) a restriction of their translation to specific tissues as suggested earlier^24–26^, we performed western blotting. In human, the *DDX3Y* testis transcripts are only translated in premeiotic male germ cells^7^.

We analyzed the translation capacity of the non-human primate *DDX3Y* transcripts in comparison to the human *DDX3Y* transcripts in three distinct tissues: testis, kidney, and liver. For this purpose, we performed immunoblotting experiments (Western blots) using the polyclonal antibodies raised against the human DBY-10 peptide^7^. The DBY-10 peptide sequence is identical in all primate specie analyzed (data not shown). Therefore, it can be assumed that these antibodies will cross react with similar intensity also to the DDX3Y proteins expressed in the tissues of these primate species.

**Figure 3:**
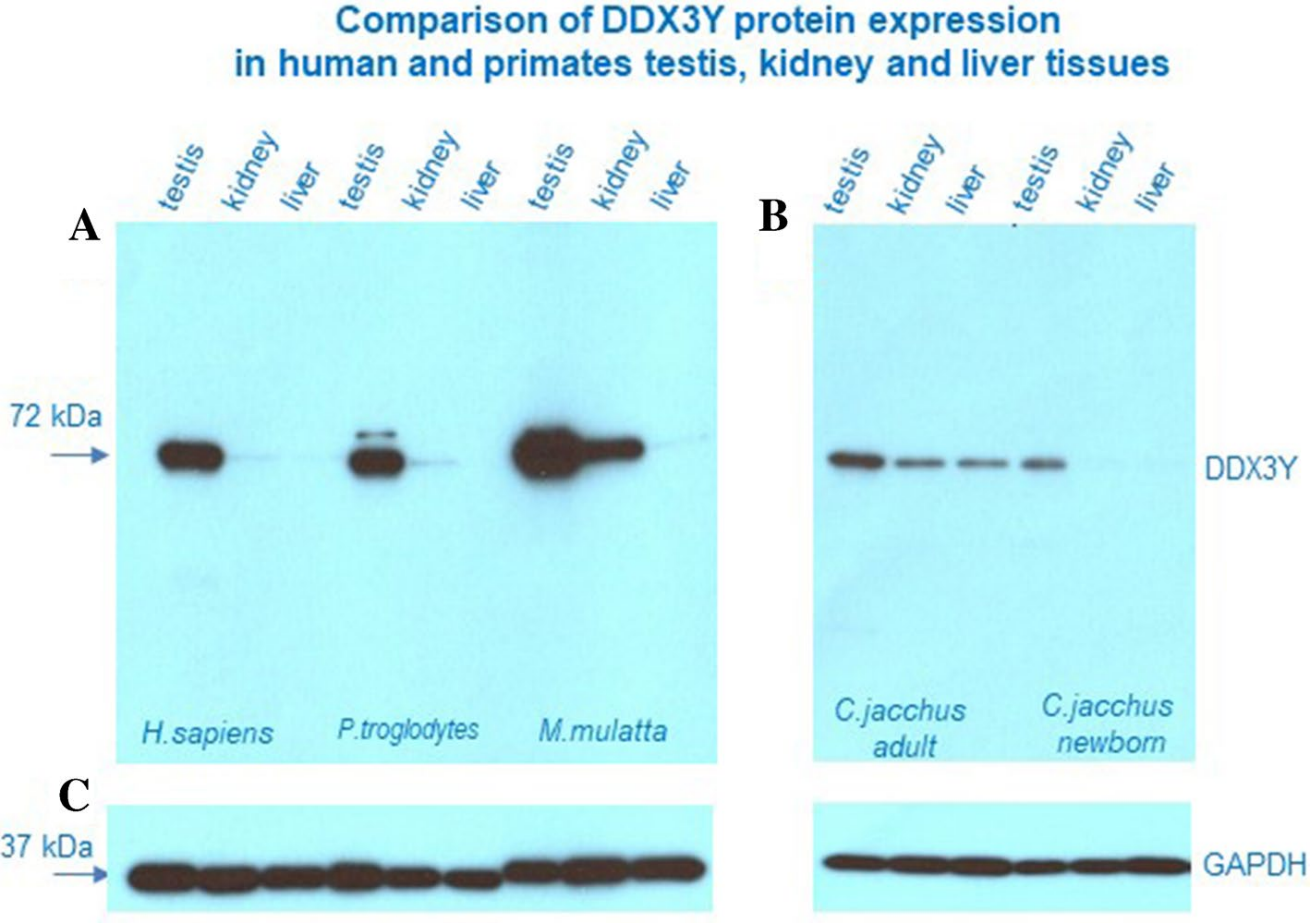
Immunoblotting experiments (Western blotting) with DBY-10, the DDX3Y-specific polyclonal antiserum^7^, are shown with protein extracts (each 10 µg) from testis, kidney, and liver tissue of human (*H.sapiens*), *Pan troglodytes*, *Macaca mulatta* and *Callthrix jacchus* in parallel. Cross-reaction with DDX3Y protein (MW = 72 kDa) was found mainly in testis tissue (10 min. exposure time). (**A**) In the old world monkey *Macaca mulatta* DDX3Y expression was also found in the kidney. (**B**) In the new world monkey, *Callthrix jacchus,* we found only, ow levels of DDX3Y expression in the *adult* and newborn monkeys. In the adult Callithix species, DDX3Y expression is found, not only in testis, but also in both somatic tissues; in the newborn phase, weak DDX3Y expression is found only in testis tissue. (**C**) Incubating the same blots subsequently with a specific antiserum against GAPDH (MW = 37 kDa) demonstrates similar sample loading in each blot lane (5 min. exposure time). Based on the comparable GAPDH intensities we conclude that strongest DDX3Y expression was found in the testis tissue of *Macaca mulatta* and testis specific DDX3Y expression only in human and *Pan troglodytes* (**A**). For improving clarity and conciseness of this Western blot presentation original blot pictures have been cropped as described in detail in Supplementary Fig. S4. Molecular Weights (MW: kDa) given at the left of the cropped blot pictures correspond to those of DDX3Y and GAPDH, respectively, as indicated on the right.

For human and *Pan troglodytes*, DDX3Y protein was found only in testis tissue (Fig. 3). In *Macaca mulatta*, strong expression of DDX3Y was found in the testis and weaker also in the kidney of this old world monkey. In *Callithrix jacchus*, DDX3Y protein expression levels are significantly lower and although expressed mainly in testis, DDX3Y was also detected in kidney and liver tissue (Fig. 3). Low DDX3Y protein expression is probably associated with the long 3´UTRs present in most *Callithrix jacchus* transcripts; they are polyadenylated at PAS4 in the distal 3´UTR (Figs. 1 and 2). When we analyzed the same tissue samples from a newborn *Callithrix jacchus* monkey, we found DDX3Y protein expression only in the testis. Our data suggest that increase of the translation capacity of the *DDX3Y* testis and kidney transcripts shown here for *Macaca mulatta* representing the catarrhine lineage, is caused mainly by shifting their polyadenylation process to PAS1 and PAS2 in the proximal 3´UTR. Lower DDX3Y protein amount in the kidney compared to testis roughly corresponds to the lower rates of PAS1 and PAS2 use for polyadenylation of the *DDX3Y* kidney transcripts (see Fig. 2). The testis specific DDX3Y protein translation in the hominoid *Pan troglodytes* is comparable to that found in human. This suggests that translation restriction to the male germ line has evolved probably first after separation of the catarrhine and hominoids lineages, i.e. ~ 23 Mya.

### Immunohistochemical analyzes of DDX3Y expression in primate testis tissue

We then wanted to analyze whether and where the DDX3Y proteins are expressed in the non human primate testis tissue and whether expression is restricted -like the human DDX3Y protein- to the premeiotic male germ cells^7^, respectively, whether the DDX3Y expression in these primates is variable as it has been shown recently for other primate spermatogenesis genes (https://www.biorxiv.org/content/10.11101/2021.11.08.467712v). For this purpose, we performed immunohistochemical staining experiments with the DBY-10 antibodies on testicular tissue sections of the marmoset monkey and two macaque species to visualize the cellular distribution of the DDX3Y protein.

We first compared expression of DDX3Y in testis tissue sections of newborn and adult *Callithrix jacchus*. The postnatal male germ cells in this new world monkey are still mainly gonocytes and pre-spermatogonia and therefore different from those found in the adult testis^29^. Only at puberty they become Ap(ale) and Ad(ark) spermatogonia like in human^30^. In parallel, we also analyzed expression of the Ki67 antigen, an indicator for dividing cells^31^ shown recently to mark proliferating male germ cells also in *Callithrix jacchus*^32^.

**Figure 4:**
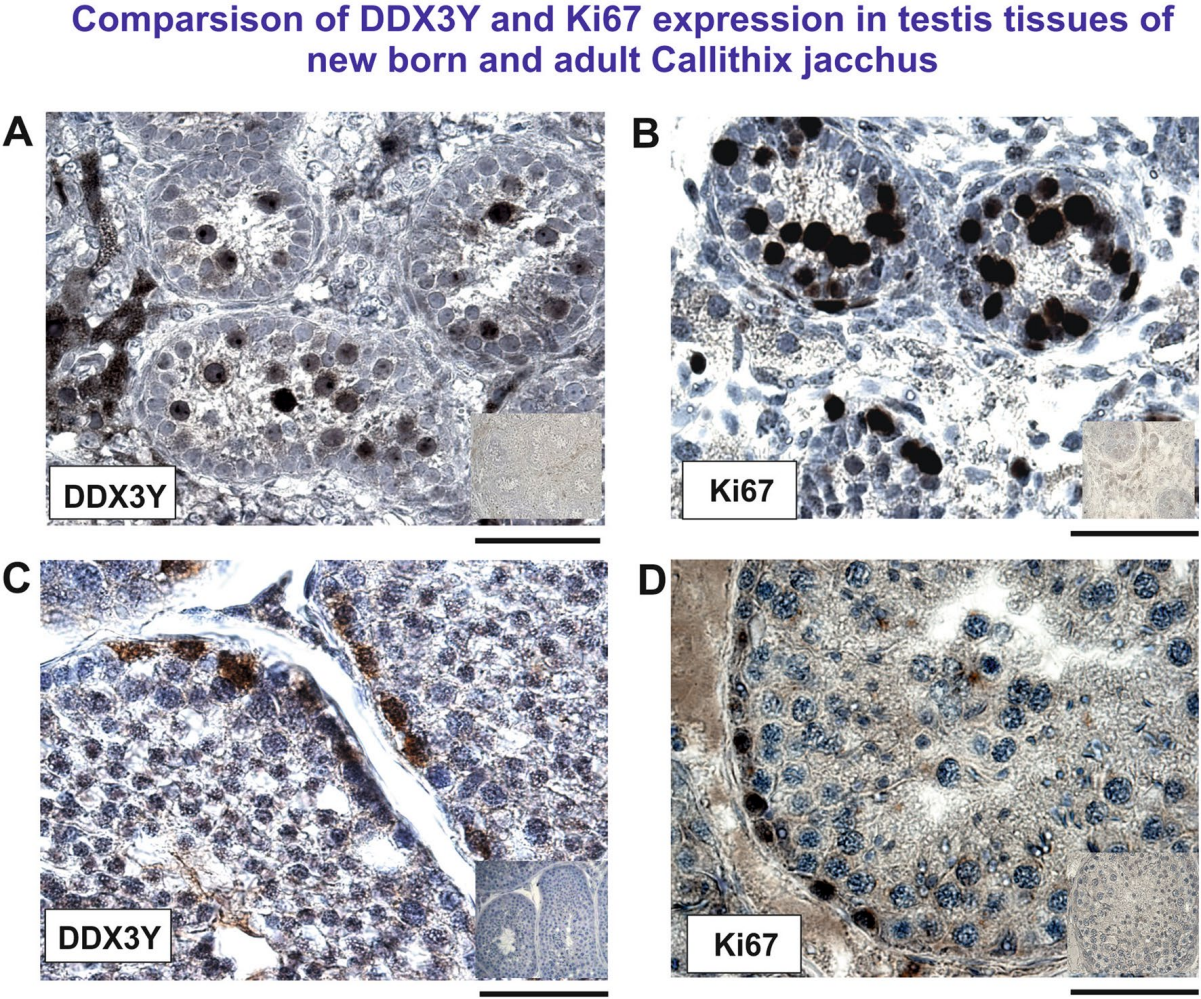
Immunohistochemical staining pattern of DDX3Y expression (**A, C**) and Ki67 expression (**B, D**) in testis tissue of newborn (**A, B**) and adult (**C, D**) *Callithrix jacchus.* It points to strong proliferation of gonocytes and pre-spermatogonia mainly located in the luminal region of the testis tubules during the new born phase of this new world monkey **(A, B)**. Adult *Callithrix jacchus* testis tissue displayed DDX3Y expression mainly in germ cells located at the basal lamina. Ki67 expression is found in the same germ cells at the basal lamina (**C, D**). Small insert pictures always at the right display immunostaining patterns with pre-immune serum on serial sections from the same testis tissues specimen. Scale bars: 50 µm.

We found strong expression of DDX3Y and Ki67 in the postnatal germ cells of all newborn *Callithrix jacchus* testes tissue sections analyzed (Fig. 4). We conclude that the gonocytes and pre-spermatogonia in the postnatal phase of spermatogenesis in this new world monkey display significant proliferation activity documented by their Ki67 expression (Fig. 4B). They are mainly located in the lumen of the testis tubules although also at the basal laminas.

Double-Immuno-Fluorescence (DIF) staining patterns of DDX3Y and Ki67 expression in testis tissue sections indicated partly overlap of both epitopes (Fig. S5). DDX3Y is thus expressed also in proliferating germ cells of this new world monkey. The DDX3Y expression pattern observed in these early postnatal male germ cells is reminiscent to that found in the human male germ cells during their prenatal phase of development^33^.

In the adult testis, DDX3Y and Ki67 expression was found in the premeiotic germ cells in this new world monkey. They seem to be mainly located at the basal lamina (Fig. 4C,D). The weak Ki67 germ cell staining pattern might have technical reasons, due to instability of the Ki67 epitope or delayed fixation when staining cells are not located at the surface of the tissue sections analyzed (R. Behr unpublished results). However, since we got similar results in different experiments, it might also reflect a low proliferation rate of the spermatogonia cells in these tissue sections. However, during the adult phase of spermatogenesis in this primate species many premeiotic spermatogonia are also apoptotically degraded^29^. This might also contribute to the observed low DDX3Y and Ki67 germ cell expression in this new world monkey. Nevertheless, some additional and independent experiments are still required to clarify the molecular etiology of this low Ki67 expression rate.

**Figure 5:**
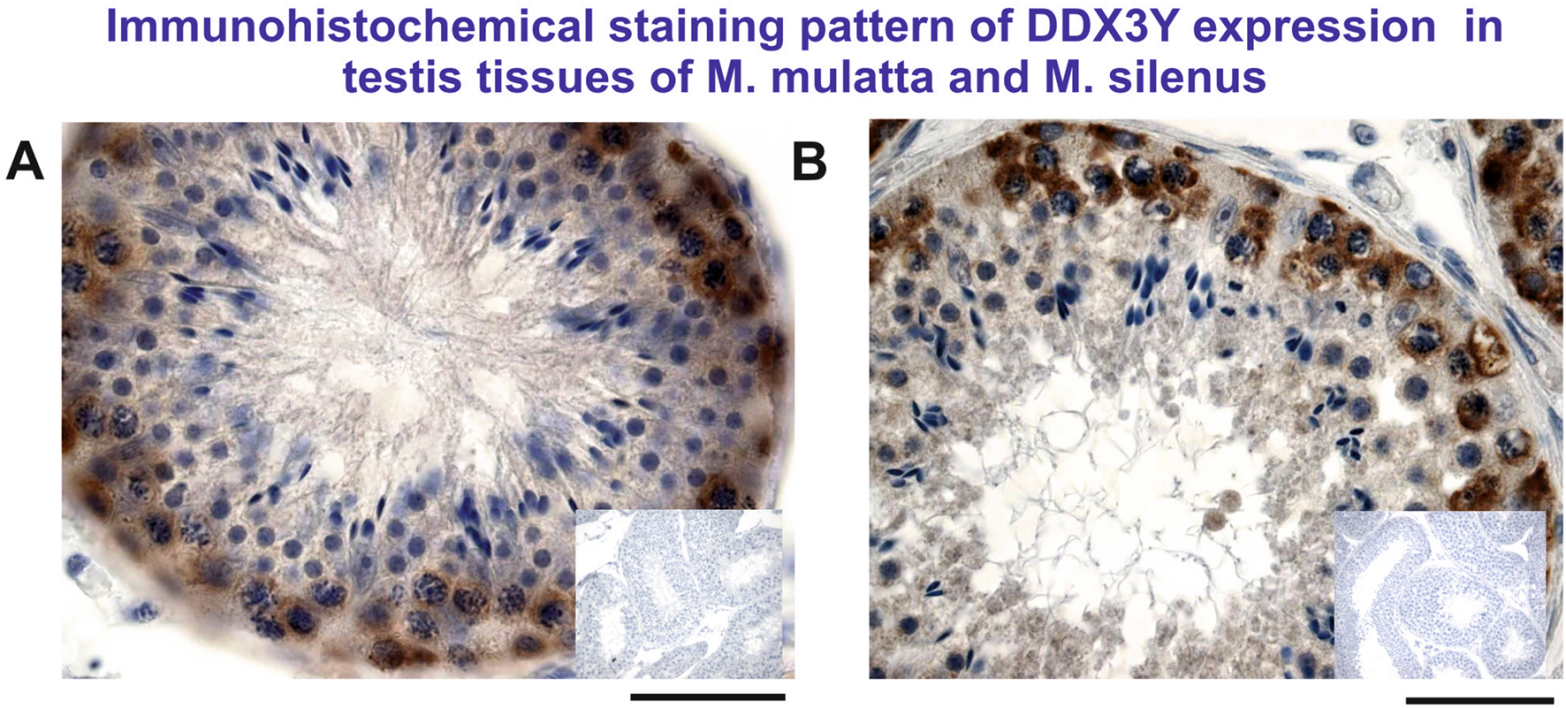
Immunohistochemical staining pattern of DDX3Y expression in testis tissue sections of *Macaca mulatta* (**A**) and *Macaca silenus* (**B**) revealed predominant and similar expression in the premeiotic male germ cells of both old world monkeys. Inserts at the right display immunostaining patterns with the pre-immune serum on overview pictures of serial sections from the same testis tissues specimen. Scale bars: 50 µm.

Strong expression of the DDX3Y protein was found in the premeiotic testicular germ cells of the macaque species: *M. mulatta* and *M. silenus* (Fig. 5). These data confirm our assumption that shift of the predominant polyadenylation process of the *DDX3Y* testis transcripts to proximal 3´UTR in the *Macaca mulatta* old world monkey (Fig. 2) has not only increased DDX3Y protein expression in the testis tissue of this catarrhine, (Fig. 3A), but also contribute to its higher and broader expression level in the premeiotic germ cells of this old world monkey when compared to that found in the premeiotic germ cells of the adult new world monkey.

We were neither able to get testis tissue sections from Pan troglodytes, nor from any somatic tissue of this primate species. However, although we cannot exclude a different DDX3Y expression in the testis tissue of this hominoid, our results shown here with both Macaque species are comparable to that found earlier in human testis^7^. A similar DDX3Y expression pattern in the premeiotic male germ cells of the testis tissue of *Pan troglodytes* is therefore expected. In summary, expression of the DDX3Y protein in testis tissue and restriction of its to the premeiotic germ cells is observed in all primates analyzed.

## Discussion

### *DDX3Y* has evolved its germ cell specific function during primate evolution

Comparing development of male germ cells in the testicular tubules of mouse and primates, it is obvious, that non-human primates are much better animal models than the mouse for exploring the basic genetic mechanisms functionally required for the complex process of male germ cell differentiation, called spermatogenesis in human^34^. This seems to be especially true for the coordinated premeiotic processes of proliferation and differentiation of spermatogonia from their stem cells^35^. It has been tried to distinguish the spermatogonial subtypes by their cellular morphology and clonal organization^36^. Molecular analyzes of a number of putative marker genes expressed only in the germ line stem cells, respectively, in germ cells during their proliferation and differentiation phase also contributed to reveal their complex heterogeneity^37^. These analyzes have indicated that there is a functional balance for these germ cells, not only to maintain their stem cell pool, but also to maintain continuous sperm production after puberty.

We believe that in non-human primates and human fine tuning of the translational control of the genes involved in this complex cellular balancing and differentiation process of spermatogonia is of utmost functional importance. Its disruption, e.g., by deletion of the *DDX3Y* gene in the AZFa interval on the human Y chromosome frequently causes complete germ cell aplasia^9^. The male germ cell specific expression of DDX3Y in the proliferating spermatogonia of the hominoids is therefore assumed to be required for this germ line specific cellular balancing process in human spermatogenesis. In this paper, we present experimental evidence that the evolutionary selection of novel 5´UTR and 3´UTR sequence elements in the *DDX3Y* gene structure of primates (see also ref. 22) has been an essential pre-condition for development of a germ cell specific translational control mechanism for *DDX3Y* expression in hominoids including human (Figs. 3, 4 and 5).

### Selection of novel functional UTR elements for *DDX3Y* gene in primate AZFa interval

First step of the *DDX3Y* genomic sequence evolution in the primate AZFa interval was probably development of the *MSY2* minisatellite sequence block on the primate Y chromosome causing the evolution of a novel *DDX3Y* promoter upstream of the conserved proximal promoter domain which became activated first and only in the male germ cells of primates^22^. The putatively second step presented in this paper, has then been the stepwise evolution of a number of novel polyadenylation sites (PASs) in the proximal 3´UTR of *DDX3Y* primate transcripts not found in mouse (supplementary informations: Fig. S1). In mouse, *Ddx3y* gene is not functional in male germ cells^21^, but a subunit of another germ cell specific translation initiation factor, namely *Eif2s3y*, is controlling spermatogonial proliferation and progression towards mouse meiosis^20^. In human, this Y gene is absent and only its X homologue, *EIF2S3X*, is still present, but is not functional in the male germ line^38^. The genomic sequence evolution in the 5´ and 3´UTR of the *DDX3Y* gene on the Y chromosome of primates can therefore be considered as a positive evolutionary selection event required for developing a novel function for the associated Y gene during the evolution of primates.

The long 5´UTRs of *DDX3Y* testis transcripts starting from the novel distal *DDX3Y* promoter in primates can modulate their translation capacity^39^. Complex secondary structures can be formed in the longest 5´UTR and a number of ATG codons upstream of the main translational ATG start codon were found to attenuate the translational capacity of these *DDX3Y* testis transcripts^39^. The functional activation of PAS2 with the canonical *“AATAAA”* sequence motif in the proximal 3´UTR of the *DDX3Y* testis transcripts in *Macaca mulatta* (Fig. 2) might have counterbalanced this attenuation effect. A significant increase of DDX3Y protein expression visible in the Western blots (Fig. 3) seems to be the functional consequence. Functional interactions between specific 5´ and 3´ termini of polyadenylated mRNAs are generally an essential control mechanism to balance their translation efficiency in distinct tissues^40,41^, and predominantly in testis tissue^42^.

### *DDX3Y* testis transcripts in hominoids are translated only in premeiotic germ cells

Comparing the *DDX3Y* transcripts translation capacity in the tissues of the primates analyzed in this study, germ cell specific translation was only observed after PAS1 was used as their major polyadenylation site occurring first in the hominoids (Figs. 2 and 3). This looks surprising because the cleavage efficiency of the PAS sequence variant, “*ATTAAA”* present in PAS1 is reported to be 30% lower^28^ than that of the canonical PAS motif, “*AATAAA”* of the neighbored PAS2.

However, it is well known, that the 3´UTR cleavage efficiency after any PAS is not only dependent on the six nucleotides of the strongly conserved PAS sequence motif *“AATAAA*” (*cis* control), but also on a number of conserved sequence elements up- and downstream of each PAS which bind more or less specifically a number of strongly conserved core proteins (*trans* control) supporting the polyadenylation process of transcripts, especially when processed in the proximal 3´UTR^43^.

In the male germ line, some of these RNA binding proteins are known to encode germ cell specific isoforms like the τCstF-64 protein expressed only in postmeiotic germ cells. Its specific germ cell function became apparent after its functional disruption causing spermatogenic defects and male infertility in mouse^44^ and human^45^.

Major candidate for a similar germ cell function in premeiotic germ cells might be the germ cell specific DAZL (Deleted in Azoospermia Like) RNA binding protein expressed in human spermatogonia^46^. It has been recently reported to control the translation of a network of genes that are critical for male germ cell proliferation^47^. Accordingly, DAZL binding was found in PAS sequence domains of thousands of mRNAs especially when located in the proximal 3´UTR. Consequently, DAZL was proposed to be functional as master regulator of a post-transcriptional mRNA program in spermatogonia essential for germ cell survival^47^. Since DDX3Y proteins are believed to be involved in the cell cycle control of proliferating spermatogonia^19^, it can be assumed that the *DDX3Y* gene transcripts might indeed be candidates for this post-transcriptional network of translation control.

If this holds true, efficient DAZL1 binding to the PAS1, but not the PAS2 sequence domain might explain the switch of predominant 3`UTR processing of the *DDX3Y* testis transcripts for polyadenylation from PAS2 to PAS1 in the hominoids. Indeed, preliminary results from analyses of the DAZL1 binding efficiency to the PAS1 and PAS2 sequence domain in the proximal 3´UTR of human *DDX3Y* transcripts indicated strong binding only to PAS1 (data not shown). However, we have not yet identified any *DAZL1* mutation in infertile man with SCO syndrome and appropriate transgenic *DAZL1* experiments in non-human primates are costly, time consuming and ethically controversial, and in human ethically inhibited. Further experiments are therefore required to test these assumptions. *DDX3Y* transcripts processed for polyadenylation at PAS1 contain long 5´UTRs including the translational repressor elements^39^. We, can therefore conclude that combining short 3´UTR lengths with long 5´UTR lengths for circle formation of *DDX3Y* transcripts might be favorable for their translation control at the ribosomes.

### DDX3Y might balance spermatogonia proliferation and differentiation in human

Proliferating male germ cells are marked for DDX3Y and Ki67 protein expression in the testis tissue of the newborn *Callithrix jacchus*; their staining pattern partly overlaps as shown by DIF staining experiments (Fig. 5S). Ki67 is a cellular marker for cell proliferation^31^. If this holds true, it would suggest that the proposed basic germ line function of DDX3Y controlling the proliferation cycle of the premeiotic spermatogonia at the G1-S phase, has already evolved in this new world monkey. However, we observed only a low translation capacity of the *DDX3Y* testis transcripts in this marmoset (Fig. 3) and suggested that this is probably due to their still predominant polyadenylation in the distal 3´UTR after PAS4 (Fig. 2).

Indeed, there is a lower number of premeiotic spermatogonial proliferation cycles in Callithrix compared to macaque^34^. Furthermore, an increased apoptotic degradation of the premeiotic spermatogonia in the testis tubules of this new world monkey has been discussed recently^32^. An increase of DDX3Y expression in the proliferating spermatogonia might thus be required first in the macaques. It has been achieved by switching the predominant polyadenylation site from PAS4 to PAS2 in the proximal 3´UTR for the *DDX3Y* testis transcripts (60%; Fig. 2).

However, this argument might not be valid for human. The number of the premeiotic proliferation cycles of spermatogonia in *Callithrix jacchus* and human are reported to be comparable^30^, i.e. lower than in the macaques Indeed the amount of DDX3Y protein in the testis of Macaca mulatta, seems to be higher than that found in the hominoids (Fig. 3).

We, therefore, speculate that activation of the distal *MSY2* promoter domain first in old world monkeys producing *DDX3Y* testis transcripts with longer 5´UTRs, than in the new world monkeys^22^ has forced an increased use of the APA sites in their proximal 3´UTR,to increase their translation capacity. It also offers probably some novel 5´UTR control elements for improving the efficiency of the spermatogonia proliferation and differentiation balancing process most likely required first in the premeiotic germ cells of the hominoids including human.

Balancing the amount of proteins functioning at the G1-S phase of the cell cycle by producing transcripts with long 5´UTRs containing complex secondary structures has been described in the literature for a number of cyclins^48^. This translation control is mandatory to inhibit the development of cancer cells showing uncontrolled proliferation. Indeed, increased DDX3Y protein expression has been reported in the pre-malignant gonadoblastoma cells and in seminoma and dysgerminoma germ cell tumors^33,49^. Increased expression of its X homologue, DDX3X, has been reported in many cancer cells originating from somatic cells^50^.

However, the DDX3 proteins can also function as tumour suppressors; they can efficiently block the proliferation cycle efficiently at the G1-S phase by e.g. activating associated repressor proteins like the p21^waf1/clp1^ protein^51^. Increased expression of DDX3Y protein in human spermatogonia might thus point to an increased demand for this balancing function of the DDX3Y protein in human spermatogonia, i.e., not only controlling the number of proliferation cycles from their stem cells, but also to ensure their further differentiation process towards mature sperm development, i.e., to maintain the life-long process of spermatogenesis in human. This might not be required in macaques, because there is a seasonal transient block of spermatogonia proliferation, which is not observed in human and not in the marmoset^34^.

In summary, in human, genome wide transcriptome analyzes have revealed a high number of gene transcripts using alternative polyadenylation (APA) mechanisms. They may enhance their cellular stability and translation efficiency, and may contribute to the evolution of novel cellular functions. Consequently, an increasing efficiency for producing distinct mRNA isoforms by the creation of novel APA sites along their 3´UTR looks like a general mechanism for ubiquitously transcribed genes like *DDX3Y* to achieve tissue specific functions^24^. Our results are in agreement with similar studies reporting a significant acceleration of evolution contributing to the complexity of testicular transcripts especially during primate evolution. These findings suggest thus positive selection mechanisms of the mammalian transcriptomes mainly in the testis organ^52^.

## Methods

### Human and primates tissue sampling

Human tissue samples used for DNA and RNA extractions were collected by our collaborating clinical partners after medical indication and written consent of the patients according to the *Declaration of Helsinki*. The study was approved by the local ethical commission of the University of Heidelberg (code: S-36-2008). Primate tissue samples were obtained from the German Primate center (DPZ; Tissue Bank of the Platform Degenerative Diseases) and the Dutch (BPRC) Primate Center via the EUPRIM network (EU contract: RII3-026,155 of the 6th Framework programme: www.euprim-net.eu). All samples were handled according to the current biosafety guidelines of the University. DNA and RNA extraction was conducted in accordance with relevant guidelines and regulations of the laboratories in the university women hospital.

#### RNA isolation and concept of nested RT-PCR assays

RNA isolations from all tissue samples were performed with the QIAGEN RNeasy Mini Kit (Cat. No. 74106) using the manufacturer’s protocol. Efficient on-column digestion of DNA during RNA purification was performed with the RNase-free DNAse digestion protocol (QIAGEN, cat. no. 79254). Concentration and purity of the mRNAs isolated were analyzed with a NanoDrop spectrometer (NanoDrop, ND-1000, USA).

First strand cDNA synthesis from the total RNA samples was performed after incubation with Oligo(dT)_15_ primer (Promega; cat. no. C1101) by reverse transcription (RT) with the Promega M-MLV Reverse Transcriptase enzyme (Cat. No. M3683) following the manufacturer’s recommendations. About 1 μg of total RNA was used in each RT reaction. All subsequent nested RT-PCR experiments were performed with the Invitrogen recombinant Taq DNA Polymerase (cat. no. 10342–020) and with 1 μl of the prepared polyA primed cDNA solution using optimal melting temperatures (usually at 61 °C).

Conventional, i.e. non-nested PCR, frequently resulted in non-specific amplification of PCR products when cycling more than 30 cycles. We therefore developed a nested PCR protocol based on a reduced number of cycles for each of the possible *DDX3Y* transcript variants: the first PCR amplification step was performed with 20 cycles. It used the so called *“outer primers*” which are located near the distinct transcriptional start sites: T-TSS-II, T-TSS-I, TSS-I(T), TSS-I-*ext* and TSS-I (*forward direction*) and upstream of the distinct PAS sites (PAS1-5) in the 3´UTRs (*reverse direction*) of the primate *DDX3Y* gene transcripts (see supplementary information: Fig. S1 and Table S1). Specific amplification products are produced only when the *DDX3Y* transcript starting at one of the selected TSS becomes polyadenylated after a PAS site located downstream of the associated reverse “*outer primers*” locations (supplementary informations; Fig. 2S). For example, transcripts polyadenylated at PAS5 will always be amplified because all *reverse* outer primers of PAS1-5 are upstream of PAS5; transcripts polyadenylated at PAS1 will only be amplified with *reverse* primers upstream of PAS1 because the *reverse* primers for PAS2-5 are located downstream of PAS1.

With 1 µl of the first PCR reaction mixture and further 20 (C. jacchus: 25) amplification cycles in the second PCR round, the cDNA PCR products became visible in a 1% agarose gel after EtBr staining only when they have started from the outer forward primers associated with the transcriptional start sites. For the second PCR round we used the so called “*inner primer*” set (supplementary information: Table S1). The common forward *“inner primer*” (# 2566; in *Callithrix jacchus*: #2663; supplementary information: Table S1) is bridging exon 16–17 and is used in each experiment. The reverse *“inner primers*” are located upstream of the PAS specific reverse “*outer primers*” (supplementary information: Fig. 3S). Complete list sequences and locations of the primer pairs used in the different RT–PCR-assays are given in Table S1. They were synthesized by Thermo Fisher Scientific (Schwerte, Germany).

### Quantitative RT-PCR experiments (TaqMan assays)

TaqMan assays were performed to compare the quantitative level of expression of *DDX3Y* transcript variants with different polyadenylation sites (PAS) in human and primate testis, kidney and liver tissues. Each real-time quantitative reverse transcription polymerase chain reaction (qRT-PCR) was performed in triplicate on a 7500 Fast Real-Time PCR System (Applied Biosystems, Germany) with a TaqMan universal PCR master mix (NoAmpErase UNG; Cat.No. 4324018; Applied Biosystems, Germany). Amplification was initiated with 10-min incubation at 95 °C for single strands formation followed by 40 cycles of 15 s at 95 °C and 1 min at 60 °C. The results were calculated using the ΔΔ*C*_T_ method and expressed as a fold-change with regard to the *DDX3Y* control transcripts through exon1-2, respectively through PAS1 (Callithrix jacchus) taken as 100%, and the *DDX3Y* transcript variants in each tissue analyzed. For normalization of the *C*_T_ values in the different experiments similar quantitative expression assays for the housekeeping genes, *HPRT (Hypoxanthine Phospho-Ribosyl-Transferase 1)* and *ACTB* (*ß-Actin)* were used accordingly. (Applied Biosystems, ABI, assays: HS99999909_m1, respectively, HS01060665_g1). Both gene expression rates were found to be constant in the different RNA pools. All TaqMan probes and F(orward)/R(everse)-primer pairs used were designed with the ABI CUSTOM TaqMan Gene expression assay software package. They are listed with Table S3. No specific TaqMan probes could be designed to distinguish polyadenylation of *DDX3Y* transcripts after PAS4, PAS5, PAS6 (only present in Callithrix jacchus) in distal 3´UTR, due to the high rate of AT nucleotides in the distal 3´UTR. This TaqMan probe has been therefore located downstream of PAS3 and upstream of PAS4 and designated as “PAS4-6” (Fig. 2). Concentration used are listed in Table S4.

### Immunoblotting

Total proteins were extracted from frozen tissue specimens using standard protocols, and resolved according to size on 12% SDS–polyacrylamide gels. PageRuler Plus Prestained Protein Ladder (10–250 kDa; Fermentas #SM1811) was used as Molecular Weight (MW) marker. Subsequently, the proteins were electrophoretically transferred from gels onto IMMOBILON-P membranes (cat.# IPVH00010; Millipore, Eschborn, Germany) and first incubated with the polyclonal DBY-10 rabbit antiserum (1:500 v/v) specifically recognising only DDX3Y proteins using the protocol as previously described^7^. A goat anti-rabbit IgG peroxidase conjugate (1:20.000 v/v; Dianova, Hamburg, Germany, cat.# 111-035-046) was used as a link antibody and visualisation of the bound antisera was done using the Western Lightning Chemiluminescence Reagent Plus kit (Perkin Elmer, cat.# NEL104001EA; Langen, Germany). Controlling similar loading of protein extracts in each gel slot incubation was performed on the same blot after DBY-10 incubation with specific GAPDH antiserum (1:2000 v/v; Santa Cruz Biotechnology, Heidelberg, Germany, cat.# sc-25778).

### Immunohistochemistry

Human and NHP testis tissue samples were fixed in buffered formaldehyde or buffered Bouin’s BOUIN´S fixative and subsequently embedded in paraffin. Tissue sections, cut 4 or 5 µm thickness, were dewaxed and rehydrated in decreasing concentrations of ethanol. Immunohistochemical staining was carried out with a standard indirect peroxidase method as described by Gueler et al.^33^. Briefly, slides were pre-treated with 0.1 M boric acid pH 7 at 60 °C overnight. After washing in permeabilization buffer (0.1 M Tris, 0.1 M NaCl, 0.1% Triton X-100; pH 7.4), endogenous peroxidase was quenched by incubation in 3% (v/v) hydrogen peroxide in methanol for 10 min at room temperature, followed by blocking with 3% (v/v) goat serum (DAKO, Glostrup, Denmark) in permeabilization buffer for 1 h. All sections were incubated overnight at 4˚C with diluted primary DBY-10 antibodies (1:500 v/v) marking specifically DDX3Y proteins^7^. Subsequently, a secondary, biotinylated goat-anti-rabbit antibody (cat.# E0432; DAKO Cytomation, Glostrup, Denmark) was applied (1:100 v/v) followed by incubation with avidin–biotin complex (VECTASTAIN ABC kit; cat.#PK6100 Vector Laboratories, Burlingame, CA, USA). Finally, slides were stained with DAB (3, 3’-diamino-benzidine tetrahydrochloride; cat.#750,118; Invitrogen, Carlsbad, CA, USA) counterstained GILL´S HEMATOXYLIN II (cat.#T864; Roth, Karlsruhe, Germany) and mounted in IMMUNO-MOUNT (cat.#0,000,402 Shandon, Pittsburgh, PA, USA).

### Double immuno-fluorescence (DIF) staining experiments

DIF staining experiments were performed on 4 μm dehydrated FFPE tissue sections. Antigen retrieval was performed by immersing the slides in 10 mM citrate buffer (pH 6.0) in a steamer for 10 min. Next, tissue sections were cooled down and kept at room temperature for 20 min. After washing the slides twice in PBS and three times in washing buffer (PBS containing 0,1% Triton X-100), they were incubated in 3% H_2_O_2_/Methanol for 10 min. After three times washing again with washing buffer, they were blocked with blocking buffer (5% normal goat serum (cat. # S-1000, Vector Laboratories, Burlingame, CA, USA) in washing buffer for 1 h at room temperature. Then tissue sections were incubated with the primary antibodies diluted in 1% goat serum/washing buffer (DBY-10: 1:500 and Ki67: 1:100 (cat.# DIA-67; OPTISTAIN, Berlin, Germany) in a humid box at 4 °C for 16 h. After three washes in washing buffer, secondary fluorescence-conjugated antibodies (Goat Anti-Rabbit DyLight 488 conjugated, cat # 35,552, and Goat Anti-Mouse DyLight 594 conjugated, cat # 35,510, Thermo Fischer Scientific, Schwerte, Germany) were diluted to a concentration of 2 µg/ml in 1% goat serum/washing buffer and incubated for 1 h at room temperature in a dark humid box. Slides were then rinsed in washing buffer five times for 3 min each and nuclei were stained with 4′,6-diamidino-2-phenylindole (DAPI, cat # D-1388, Sigma) at a concentration of 1 μg/ml for 10 min at RT. Sections were then rinsed three times with water for 3 min each before being mounted and coverslipped using ProLong Glass Antifade Mountant (cat.#P36980, Invitrogen).The imaging was performed using a fluoreszenz microscope (LEICA) with magnification 100x.

### In silico* analyzes of nucleic sequences*

Sequence comparisons and homology searches were performed by MegaBLAST and BLASTN via the BLAST server at NCBI. Multiple sequence alignments were performed using CLUSTAL W2, release 2.0.10, through the EMBL-EBI web server (http://www.ebi.ac.uk).

## Supplementary Information

Supplementary Information.

## Data Availability

All data generated or analysed during this study are included in this published article and its Supplementary Information files.

## References

[1] Vogt PH (1998). Human chromosome deletions in Yq11, AZF candidate genes and male infertility: History and update. Mol. Hum. Reprod..

[2] Vogt PH (2005). Azoospermia factor (AZF) in Yq11: Towards a molecular understanding of its function for human male fertility and spermatogenesis. Reprod. BioMed. Online.

[3] Vogt PH, Edelmann A, Kirsch S, Henegariu O, Hirschmann P, Kiesewetter F (1996). Human Y chromosome azoospermia factors. (AZF) mapped to different subregions in Yq11. Hum. Mol. Genet..

[4] Luddi A, Margollicci M, Gambera L, Serafini F, Cioni M, De Leo V (2009). Spermatogenesis in a man with complete deletion of *USP9Y*. N. Engl. J. Med..

[5] Krausz C, Degl'Innocenti S, Nuti F, Morelli A, Felici F, Sansone M (2006). Natural transmission of USP9Y gene mutations: A new perspective on the role of AZFa genes in male fertility. Hum. Mol. Genet..

[6] Vogt PH, Ditton HJ, Kamp C, Zimmer J, Lau YF, Chan WY (2007). Structure and function of AZFa locus in human spermatogenesis. The Y Chromosome and Male Germ Cell Biology in Health and Diseases.

[7] Ditton HJ, Zimmer J, Kamp C, Rajpert-De Meyts E, Vogt PH (2004). The AZFa gene *DBY (DDX3Y)* is widely transcribed but the protein is limited to the male germ cells by translation control. Hum. Mol. Genet..

[8] Foresta C, Ferlin A, Moro E (2000). Deletion and expression analyzis of AZFa genes on the human Y chromosome revealed a major role for DBY in male infertility. Hum. Mol. Genet..

[9] Kamp C, Huellen K, Fernandes S, Sousa M, Schlegel PN, Mielnik A (2001). High deletion frequency of the complete AZFa sequence occurs only in men with complete germ cell aplasia (Sertoli-cell-only-syndrome). Mol. Hum. Reprod..

[10] Tyler-Smith C, Krausz C (2009). The Will-o`-the wisp of genetics- hunting for the azoospermia factor gene. N. Engl. J. Med..

[11] Tiepolo L, Zuffardi O (1976). Localization of factors controlling spermatogenesis in the nonfluorescent portion of the human Y chromosome long arm. Hum. Genet..

[12] Krausz C, Casamonti E (2017). Spermatogenic failure and the Y chromosome. Hum. Genet..

[13] Lahn BT, Page DC (1997). Functional coherence of the human Y chromosome. Science.

[14] Chang T-C, Liu W-S (2010). The molecular evolution of PL10 homologs. BMC Evol. Biol..

[15] Chuang RY, Weaver PL, Liu Z, Chang TH (1997). Requirement of the DEAD-Box protein ded1p for messenger RNA translation. Science.

[16] Soto-Rifo R, Ohlmann T (2013). The role of the DEAD-box RNA helicase DDX3 in mRNA metabolism. WIREs RNA.

[17] Lai MC, Chang WC, Shieh SY, Tarn WY (2010). DDX3 regulates cell growth through translational control of cyclin E1. Mol. Cell Biol..

[18] Sekiguchi T, Iida H, Fukumura J, Nishimoto T (2004). Human DDX3Y, the Y-encoded isoform of RNA helicase DDX3, rescues a hamster temperature-sensitive ET24 mutant cell line with a DDX3X mutation. Exp. Cell Res..

[19] Ramathal C, Angulo B, Sukhwani M (2015). *DDX3Y* gene rescue of a Y chromosome AZFa deletion restores germ cell formation and transcriptional programs. Sci. Rep..

[20] Mazeyrat S, Saut N, Grigoriev V, Mahadevaiah SK, Ojarikre OA, Rattigan A (2001). A Y-encoded subunit of the translation initiation factor Eif2 is essential for mouse spermatogenesis. Nat. Genet..

[21] Matsumura T, Endo T, Isotani A, Ogawa M, Ikawa M (2019). An azoospermic factor gene, *Ddx3y* and its paralog, *Ddx3x* are dispensable in germ cells for male fertility. J. Reprod. Dev..

[22] Rauschendorf MA, Zimmer J, Hanstein R, Dickemann C, Vogt PH (2011). Complex transcriptional control of the AZFa gene *DDX3Y* in human testis. Int. J. Androl..

[23] Bao W, Zhu S, Pandya A, Zerjal T, Xu J, Shu Q (2000). MSY2: A slowly evolving minisatellite on the human Y chromosome which provides a useful polymorphic marker in Chinese populations. Gene.

[24] Lianoglou S, Garg V, Yang JL, Leslie CS, Mayr C (2013). Ubiquitously transcribed genes use alternative polyadenylation to achieve tissue-specific expression. Genes Dev..

[25] Mayr C (2016). Evolution and biological roles of alternative 3´UTRs. Trends Cell Biol..

[26] MacDonald CC (2019). Tissue-specific mechanisms of alternative polyadenylation: Testis, brain, and beyond (2018 update). WIREs RNA.

[27] Vong QP, Li Y, Lau Y-FC, Dym M, Rennert OM, Chan W-Y (2006). Structural characterization and expression studies of *Dby* and its homologs in mouse. J. Androl..

[28] Sheets MD, Ogg SC, Wickens MP (1990). Point mutations in AAUAAA and the poly(A) addition site: Effects on the accuracy and efficiency of cleavage and polyadenylation in vitro. Nucleic Acids Res..

[29] Li L-H, Donadl JM, Golub MS (2005). Review on testicular development, structure, function, and regulation in common marmoset. Birth Defects Res. Part B.

[30] Millar MR, Sharpe RM, Weinbauer GF, Fraser HM, Saunders PT (2000). Marmoset spermatogenesis: Organizational similarities to the human. Int. J. Androl..

[31] Gerdes J, Lemke H, Baisch H, Wacker HH, Schwab U, Stein H (1984). Cell cycle analyzis of a cell proliferation-associated human nuclear antigen defined by the monoclonal antibody Ki-67. J. Immunol..

[32] Lin Z, Yu C, Hirano T, Shibata S, Seki NM, Kitajima R, Sedohara A (2015). Gene expression ontogeny of spermatogenesis in the marmoset uncovers primate characteristics during testicular development. Dev. Biol..

[33] Gueler B, Sonne SB, Zimmer J, Hilscher B, Hilscher W, Graem N (2012). AZFa protein DDX3Y is differentially expressed in human male germ cells during development and in testicular tumours: New evidence for phenotypic plasticity of germ cells. Hum. Reprod..

[34] Behr R (2017). Characteristic features of male germline development in primates, Genetics of human infertility PH Vogt. Monogr. Hum. Genet..

[35] Boitani C, Di Persio S, Esposito V, Vicini E (2016). Spermatogonial cells: Mouse, monkey and man comparison. Semin. Cell Dev. Biol..

[36] Ehmcke J, Schlatt S (2006). A revised model for spermatogonial expansion in man: Lessons from non-human primates. Reproduction.

[37] Fayomi AP, Orwig KE (2018). Spermatogonial stem cells and spermatogenesis in mice, monkeys and men. Stem Cell Res.

[38] Ehrmann IE, Ellis PS, Mazeyrat S, Duthie S, Brockdorff N, Mattei MG (1998). Characterization of genes encoding translation initiation factor eIF-2gamma in mouse and human: Sex chromosome localization, escape from X-inactivation and evolution. Hum. Mol. Genet..

[39] Jaroszynski L, Zimmer J, Fietz D, Bergmann M, Kliesch S, Vogt PH (2011). Translational control of the AZFa gene DDX3Y by 5'UTR exon-T extension. Int. J. Androl..

[40] Gallie DR (1998). A tale of two termini: A functional interaction between the termini of an mRNA is a prerequisite for efficient translation initiation. Gene.

[41] Hughes T (2006). Regulation of gene expression by alternative untranslated regions. Trends Genet..

[42] Denoeud F, Kapranov P, Ucla C, Frankish A, Castelo R, Drenkow J (2007). Prominent use of distal 5´ transcription start sites and discovery of a large number of additional exons in ENCODE regions. Genome Res..

[43] Tian B, Manley JL (2017). Alternative polyadenylation of mRNA precursors. Nat. Rev. Mol. Cell Biol..

[44] Dass, B., Tardif T., Park J. Y., Tian B., Weitlauf H. M., Hess R. A., Carnes K., Griswold M. D., Small C L. & MacDonald C. C. Loss of polyadenylation protein τCstF-64 causes spermatogenic defects and male infertility. In *Proceedings National Academy of Science, U.S.A.* **104**, 20374–20379 (2007). 10.1073/pnas.0707589104 PMC2154438 18077340

[45] Dass B, McDaniel L, Schultz RA, Attaya E, MacDonald CC (2002). The Gene *CSTF2T*, Encoding the human variant CstF-64 polyadenylation protein cCstF-64, lacks introns and may be associated with male sterility. Genomics.

[46] Vangompel MJW, Xu EX (2011). The roles of the DAZ family in spermatogenesis: More than just translation?. Spermatogenesis.

[47] Zagore LL, Sweet TJ, Hannigan MM, Weyn-Vanhentenryck SM, Jobava R, Hatzoglou M (2018). DAZL regulates germ cell survival through a network of polya-proximal mRNA interactions. Cell Rep..

[48] Tarn W-Y, Lai M-C (2011). Translational control of cyclins. Cell Div..

[49] Vogt PH, Besikoglu B, Bettendorf M, Frank-Herrmann P, Zimmer J, Bender U (2019). Gonadoblastoma Y locus genes expressed in germ cells of individuals with dysgenetic gonads and a Y chromosome in their karyotypes include DDX3Y and TSPY. Hum. Reprod..

[50] Mo J, Liang H, Su C, Li P, Chen J, Zhang B (2021). DDX3X: Structure physiologic functionas and cancer. Mol. Cancer.

[51] Chao C-H, Chen C-M, Cheng P-L, Shih J-W, Tsu A-P, Lee Y-HW (2006). DDX3, a DEAD Box RNA helicase with tumour growth suppressive property and transcriptional regulation activity of the *p21*^*waf1/clp1*^ promoter, is a candidate tumour suppressor. Cancer Res..

[52] Khaitovich P, Enard W, Lachmann M, Pääbo S (2006). Evolution of primate gene expression. Nat. Rev. Genet..

