# Supplementary material for: AZFa Y gene, DDX3Y, evolved novel testis transcript variants in primates with proximal 3´UTR polyadenylation for germ cell specific translation
Source: Sci Rep. 2022 May 27;12:8954. doi: 10.1038/s41598-022-12474-0 (PMC9142519; doi:10.1038/s41598-022-12474-0)
Supplement: Supplementary file 1 — Supplementary Information. [file 41598_2022_12474_MOESM1_ESM.pdf]

Fig. 1S Vogt et al.

Comparative map of putative polyadenylation sites (PASs) with canonical “AATAAA” motif (i.e., 100% cleaving efficiency) and “ATTAAA” motif variant with somewhat lower cleavage efficiency dependent on tissue [27] along the *DDX3Y* 3´UTR sequences of human (Hs), Pan troglodytes (Pt), Macaca mulatta (Mmul), Callitrix jacchus (Cj), and Mouse musculus (Mm) using CLUSTAL multiple sequence alignment tool (see Material & Methods). GenBank accession numbers are as follows: Hs: AC004474; Pt: AC146254; Mmul: AC213321; Cj: AC225609; Mm: AC006508. They all start with the conserved TGA translation stop codon (marked grey) in exon 17, here sequence #1. Unique mouse PAS sites are distinguished by red colour code. Only the last of the 11 putative mouse PAS sites (PAS11) is conserved in primates *DDX3Y* 3´UTR (PAS5; Cj.:PAS6).

```
Hs              TGAATCTGCTTTGCAGCAAAGTCACCCTTACA---AAGAAGCTAATATGGAAACCACATG 57
Pt              TGAATCTGCTTTGCAGCAAAGTCACCCTTACCTTTAAGAAGCTAATATGGAAACCACATG 60
Mmul            TGAATCTGCTTTGCAGCAAAGTCACCCTTACA---AACAAGCTAATATGGAAACCACATG 57
Cj              TGAATCTGCTTCGCAGCAATGTCACCCTTACA---AACAAGCTAATATGGAAACCACGTG 57
Mm              TGAA-----TTTGTATCA---TTA--TTT-CA---TACA-GCTAATATGGAAACCACATG 45
                ****     ** * * **   * *   ** *     * * ***************** **

Hs              TAACTTAGCCAGACTATATTGTGTAGCTTCAAGAACTTGCAGTACATTACCAGCTGTGAT 117
Pt              TAACTTAGCCAGACTATATTGTGTAGCTTCAAGAACTTGCAGTACATTACCAGCTGTGAT 120
Mmul            TAACTTAGCCAGACTGTATCGTGTAGCTTCAAGAACTTGCAGTACATTACCAGCTATGAT 117
Cj              TAACTTAGCCAGACTGTGTCGTGTAGCCTCAAGAACTTGCAGGACGTTACCAGCAGTGAT 117
Mm              TAACTTAGCCAGACTGTATTGTGTAGCTTCAAGAACTTGCAGTACATTACCAGCTGTGAT 105
                *************** * * ******* ************** ** ********  ****

                                                                  Mmul + Cj PAS1
Hs              TCTCCTGATAATTCAAGGGAGCTCAAAGTCACAAGAAGAAAAATGAAAGGAAA--AAACA 175
Pt              TCTCCTGATAATTCAAGGGAGCTCAAAGTCACAAGAAGAAAAATGAAAGGAAA--AAACA 178
Mmul            TCTCCTGATAATTCAAGGGAGCTCAAAGTCACAAGAAGAAAAATGAAAGGAAT--AAACA 175
Cj              TGTCCTGAAAATTCAAGGGAGCTCAAAGTCACAAGAAGAAGAATGAAAGGAAATAAAACA 177
Mm              TCTTCTAAAAGTTCATGAGAGCTCAAAGTCACAAGAA----ATT--AAGCAAC--AACCT 157
                * * ** * * **** * *******************    * *  *** **   ** * 

                                                               Hs + Pt      PAS1
Hs              GCAGCCCTATTCAGAAATTGGTTTGAAGATGTAATTGCTCTAGTTTGGATTAAACTCTTC 235
Pt              GCAGCCCTATTCAGAAATTGGTTTGAAGATGTAATTGCTGTAGTTTGGATTAAACTCTTC 238
Mmul            GCAGCCCTATTAAGAAATTGGTTTGAAGATGTGATTGCTATAGTTTGGATTTAACTCTTC 235
Cj              GCAGCCCTATTCAGAAATTGGTTTGAAGACTTAATTGCTGTGATTTGGATTTAACTCT-C 236
Mm              GTAGCCCTATTCA-AAGTTGAT--GAGAA-A-AATTTTTGTAACT-GGATT-AA-T-T-C 207
                * ********* * ** *** *  **  *    ***  * *   * ***** ** * * *

Hs              CCCTCCTGCTTTAGTGCCACCCCAAACTGCATTTATAATTTTGTGACTGAGGATCGTTT- 294
Pt              CCCTCCTGCTTTAGTGCCACCCCAAACTGCATTTATAATTTTGTGACTGAGGATCGTTT- 297
Mmul            CCCTCCTGCTTTATTCCCACCCCAAACTGCATTTATAATTTTGTGACTGAGGATCGTTC- 294
Cj              CCCTCCTGCTTTAGTCCCACCCCAAACTGCATTTATAAGTTTGTGGCTAAGGATCGTTTT 296
Mm              ACTTCCTGCTT-GGTTGTACCCTAAACAGCATTTATAA-TTTGTGAGTGAGGATCATT-- 263
                 * ********   *   **** **** ********** ******  * ****** **  

Hs              GTTTGTTAACGTACTGTGACTTT-AACTTTAGACAACTTA-CTACTTTGATGTCCTGTTG 352
Pt              GTTTGTTAACGTACTGTGACTTT-AACTTTAGACAACTTAACTACTTTGATGTCCTGTTG 356
Mmul            GTTTGTTAACGTACTGTGACTTT-AACTTTAGACAACTTA-TTACTTTGATGTCCTTTTG 352
Cj              GTTTGTTAACATACTGTGACTTT-AACTTTAGACAGCTTC-CTACTTTGATGTCCTGTTG 354
Mm              ---TGTTAATGTACTATGACTTTTAACTTTAAACAACTT----A-TTTAATGTCCTGTTG 315
                   ******  **** ******* ******* *** ***    * *** ******* ***

                                                    Hs + Pt + Mac + Cj      PAS2
Hs              GCTCAGTAATGCTCACGATACCAATTGTTTTGACAAAA-TAAATTTACTAAACTTGGCCT 411
Pt              GCTCAGTAATGCTCATGATACCAATTGTTTTGACAAAA-TAAATTTACTAAACTTGGCCT 415
Mmul            GCTCAGTAATGCTCACGATACCAATTGTTTTGACAAAAATAAATTTACTAAACTTGGCCT 412
Cj              GATCAGTCATGCTCACGATACCCACTGTTTTGACAAAA-TAAATTTACTAACCTCGGCCT 413
Mm              TCTCAATAATGCTCAAGATATAAATTGTTTTAATAAGT-TAA--TTG--AATTTTGGC-T 369
                  *** * ******* ****   * ****** * **   ***  **   **  * *** *

Hs              AAAATCAAACCTTGGCACAGAGGTATGATACAACTTTAACAGGAGTCATCAATTCATC-C 470
```

```
Pt      AAAATCAAACCTTGGCACAGAGGTATGATACAACTTTAACAGGAGTCATCAATTCATC-C 474
Mmul    AAAATCAAACCTTGGCACACAGGTATGATACAACTTTAACAGGAATCATCAATTCATC-C 471
Cj      AAAATCAAACCGTGGCACACAGGTGTGATACAACTTCAACAGGAATCATCAATTCATC-C 472
Mm      ACAAGCAAACCTTGGCACACAAGTGTAATACA-CCTTAATAGGA---ATCAATTCATTGC 425
        * ** ****** ******* * ** * ***** * * ** ****   **********  *

                                                          Mm    PAS1
Hs      ATAAATATAAA-AAGGGAAAAA-AA----------CTTAAGGCAGTAGTCTGCATTA-GG 517
Pt      ATAAATATAAA-AAGGAAAAAA-AA----------CTTCAGGCAGTAGTCTGCATTA-GG 521
Mmul    ATAAATATAAA-AAGGAAAAAA-AA----------CTTCAGGCAGTAGCCTGCGTTA-GG 518
Cj      GTGAATATAAACGGGGAAAAAACAAAACAAAACATCTTCAGACAGTAGCCTGCATTC-GG 531
Mm      -TAAGTATGAT---GGTGAATG-AA----------CTT-A-----T--CCTGGATAATAA 462
         * * *** *     **  **   **          *** *     *   ***  *

                                            Hs + Pt + Mac + Cj PAS3
Hs      ACTGTTTGAGTTTTGCAGACTTGG-GG-TTGGGAGAACATCTTAAAGCATTAAAGCATAG 575
Pt      ACTGTTTGAGTTTTGCAGACTTGG-GG-TTGGGAGAACATCTTAAAGCATTAAAGCATAG 579
Mmul    ACTGTTGGATTTTTGCAGACTTGG-GGGTTGGGAGAACATCTTAAAGCATTAAAGCATAG 577
Cj      ACCGCTTGACTCTTGCAGTCTTGGCGG-GTGGGGGATCATCTTAAAGCATTAAAGCATAG 590
Mm      ATT-TTTGGTATATGCAGAGTTG-----TTGGTAAAACTTCTT----T-TT----TA-AG 506
        *    * *     *****  ***       ***    * * ****       **      * **

Hs      TTT-TTT--------GTATGGCCAACCT-TA---CTAAATTAAGTTCTGACTTGCTCACT 622
Pt      TTT-TTT--------GTATGGCCAACCT-TA---CTAAATTAAGTTCTGACTTGCTCACT 626
Mmul    TTT-TTT--------ATATGGCCAACCTGTA---CTAAA----GTTCTGACTTGCTCACT 621
Cj      TTTGTTTTTTTTTTAATGTGACCAACCTATA---GTAA-----GTTCTGATTTTCTCAGT 642
Mm      TTTGTA---------ATGTGGCAGACATACAAAACTAAA----GTTCTGAACTGCTCACT 553
        *** *           * ** *  ** *  *    ***      *******  * **** *

Hs      CTATCCTGGATAGGCACTTGGGAACTTACACTCTTTAA----GCCATTCCAGTCATGATG 678
Pt      CTATCCTGGATAGGCACTTGGAAACTTACACTCTTTAA----GCCATTCCAGTCATGATG 682
Mmul     CTATCCTGGATAGGCACTTAGAAACTTACACTCTTTAA----GCCATTCCAGTCATGACG 677
Cj      GTATCCTGGAGGGGCACTTAGAAACTTACCCTCTTTAAATAAGCCATTCCAGTGGTGAGG 702
Mm      ATATCCTGCATTGACACTTAGAACCTTA---------AA----GT-ATTA-AAT-AT-ATG 597
         ******* *  * ***** * * ****          **     *   ***   * *   * * *

Hs      AGGTGGAATGTATCAGTATACCAATTAATATTTTTGAAAGAGCTCTTTTAGGTTAATTTA 738
Pt      AGGTGGAATGTATCAATATACCAATTAATATTTTTGAAAGAGCTCTTCTAGGTTAATTTA 742
Mmul    AGGTGGAATGTATCAATATACCCATTAATATTTTTGAAAGAGCTCTTTTAGGTTAATTCA 737
Cj      AGGTGGAATGTATCACCCTACCCATTAATATTTTTGAAAGAGCTCTTA-AAGTTAACTGA 761
Mm      A--TT--ATATATGGTTATATCC-TT--TATTTTC-AAA-AGC------A--CTAATGCA 640
        *  *   ** ***     ** *  **  ******  *** ***       *   ***   *

Hs      AGTA----CAGCAATTTCTCATGTAATGTTTAGGGAGTTTATTCTAACCTAGGCAA--AC 792
Pt      AGTAAGTACAGCAATTTCTCATTTAATGTTGAGGGAGTTTATTCTAACCTAGGCAA--AC 800
Mmul    AGTAAATACAGCAATTTCTCATTTAATGTTTAGGGAGTTTATTCTAAGCTAGGCAACTGT 797
Cj      GGTAAATACAGCAGTTTCTCTTTTAAAGTTTGAGAAGTTTATTCTAAGCTAGGCAAACGT 821
Mm      GATAACTACAGTGATTCCTCATTTACTGTTTTA--AGTA-ATT-TA---TA--T-----T 686
          **     ***    ** *** * **  ***      ***  *** **    **

Hs      GG-CATGCTATCACAAGAAAGGTT----TAAAGCTTTGAT-AA-AATG---GGGGAGATT 842
Pt      GG-CATGCTATCACAAGAAAGGTT----TAAAGCTTTGAT-AA-AATG---GGGGAGATT 850
Mmul    GGGCATACTATCACAGGAAAGATT----TAAAGCTTTGAT-AC-AATG---GGGGAGATT 848
Cj      GGGCATACTGTCACAGGAAAGGTTGGTTTAAAGCTTTGAT-AA-GATGTTTGGGGAAATA 879
Mm      GG-CATACTCCTACATGAAAG-TT----TAAAATTTTGGTGAAGAATT---TGGGATATA 737
        ** *** **     *** ***** **      ****  **** * *    **      **** **

Hs      TAATC--AGT--TTTTTTAATGCCTGCTATAAA-AATTTGAAATATTAGA--A-TGGCCG 894
Pt      TAATC--AGT--TTTTTTAATGCCTGCTATAAA-AATTTGAAATATTAGA--A-TGGCCG 902
Mmul    TCAT---AGT--TTTTTTAATGCCTGCTATAAA-AATTTGAAATGTTAGA--A-TGGCTG 899
Cj      TCATTTAAGTAGTTTTCTTATGCCTGCTGTAAA-AATTCAAAATGTTAGA--A-TGGCTG 935
Mm      AAGTTTAGGT--ATATTTAATGCCTGCCATGCATAA---AAAATGGTAAATTACTAGGTG 792
            *     **    * * * ********  *   * **     ****  ** *   * * *   *

Hs      ACCATGGCAGTGACCAGGCCTCACTACAGGCCTGGTTGGATTCTGGTCTTTAA-TGCATG 953
Pt      ACCATGGCAGTGACCAGGCCTCACTACAGGCCTGGTTGGATTCTGGTCTTTAA-TGCATG 961
Mmul    ACCATGGCAGTGACCAGGCCTCACTATAGGCCTGGTTGGATTCTGGTCTTTTA-TGCATG 958
Cj      GCCATGGCAACGACCAAGTCTCACGGCAGGCCTGATTGGTTTCTGGTCTTTAACTGCATG 995
Mm      ACTATGGCAATGACCAGACCTTATTA-AAGCTTTGTTA-ATTCTGGTCTTTAA-TGCATG 849
         * ******  *****    ** *     * ** *   **    *********** * ******

Hs      CTAGTGTTGATGTTTTTTGGTCAAGAACGGT-TTAAACAGGAAGGAT-TGTGCAGCAGGC 1011
Pt      CTAGTGTTGATGTTTTTTGGTCAAGAATGGT-TTAAACAGGAAGGAT-TATGCAGCAGGC 1019
Mmul     CTAGTGTTGATGTTTTTTGGTCAAGAAGGGT-TTAAACAGGAAGGAT-TATGCAGCAGGC 1016
Cj      CTAGTGTTTATGTTCTTCAGTCAAGAAGGAAATTAAACAGGAAGGAT-TATGCAGCAGGC 1054
```

```
Mm         CTAGTGTTGGTGTTTTTTGGTCAAGA-GAGT-ATGAACAG--ATTATGCA-GCAGCAGGC 904
           ********  **** **  *******       * *****  *  **     *********
                                                           Mm     PAS2-5
Hs         TTTAATTT-A-A-TGT-AGATT---C-ATACTG-CTCTGTTAAAGC-TGCAT-TGAAATG 1060
Pt         TTTAATTT-A-A-TGT-AGATT---C-ATACTG-CTCTGTTAAAGC-TGCGT-TGAAATG 1068
Mmul       TTTAATTT-A-A-TGT-AGAGT---C-ATATTG-CGCTGTTAAAGC-TGCAT-TGAAATG 1065
Cj         TTTAATGT-A-AATGT-AGATT---A-GTAATG-CTCTGTTGTAGC-TGCAT-TGAAATG 1104
Mm         TTTACTTTTACACAGTCAGGTTTTTCTGTAACAACTATGTTGAAATGTAAAAATAAAATA 964
           **** * * * *  ** **  *       **    *  ****  *   *     * ****

                                                        Mm        PAS6
Hs         T--TAAAAT------GGCTTACACTTGCAGACTTTGCAAATCTTAAGACTAACAAATC-- 1110
Pt         T--TAAAAT------GGCTTACACTTGCAGACTTTGCAAATCTTAAGACTAACAAATC-- 1118
Mmul       T--TAAAAT------GGCTTACACTTGTAGACTTTGTAAAGCT-GAGACTAACAAATT-- 1114
Cj         T--TAAAAT------GGCTTACACTTGTGGACTTTTTAAATCT-AAGGCTGCCAAATG-- 1153
Mm         AAATAAAATAAAAAAGGCTTACA--TACATACTATGTAAATAT-AAGAATAAAAATTGA 1021
              ******      ********  *     *** *  ***  *  **  *   ****

Hs         --CT--TGAAATCACACAGCTTGCAAATACGTACTAAACTGCACAAGGTGTGTGTTCTAT 1166
Pt         --CT--TGAAATCACACAGGTTGCAAATACGTACTAAACTGCACAAGGTGTGTGTTCTAT 1174
Mmul       --CT--TGATATCACACAGATTGCAAATATGTACTAAACTGCACAAGGTGTGTATGCTCT 1170
Cj         --CT--TGAAATCACACAGGTTGCATTTATGTACTAAACTGCACAAGGTATACCTTCTCT 1209
Mm         AATTGCTGAGATCTCACAGGTTGGATTTATGTACTAA-TTATACATGATG-GTCAG-TAC 1078
              *  *** *** ***** *** *  ** *******  *  *** * *        *

Hs         ATGTGCAGTTTTAGCGTATTTTAGTTGCATAGGTTTCCATGGTATTTATAGTC-TCTTGT 1225
Pt         ATGTGCAGTTTTAGCGTATTTTAGTTGCATAGGTTTCCATGGTATTTATAGTC-TCTTGT 1233
Mmul       ATGTGCAGTTTTAGTGTATTTTAGTTGCATAGGTTTCCATGGTATTTATAGTC-TCTTGT 1229
Cj         -C-TGCAGTTTTAGTGTATTTTAGTTGCATAGGTTTCCATTGTATTAGCAGTC-TCTTGT 1266
Mm         A-GTGCAGTTTTCATCTATTTTCGTTGCATAGGTTTCCATTGTATT-ATAGTCATATT-T 1135
              *********    ****** ***************** *****    **** * ** *

Hs         GCTAAATTTGGCCAAAGATGATTGTCCACCACTAAAAATGCCTCTCCCACTTG-GAATTC 1284
Pt         GCTAAATTTGGCCAAAGATGATTGTCCACCACTAAAAATGCCTCTCCCACTTG-GAATTC 1292
Mmul       GCTAAATTTGGCCAAAGATGATTGTCCACCACTAAAAATGCCTCTCCCACTTG-GAATTC 1288
Cj         GGTAAATTTGGCCAAAGACGGTTGTCCACCGCTCAAAATGCCTCTCTCACTTG-GAATTC 1325
Mm         --TAA--TTGGCCAAAAATGATTGTCCCTCACAAAAAATGCCTTTTCC-TTTGAGAATT- 1189
             ***  ********* * * ******   * *  ********* *  *   *** *****

                                     Mm                              PAS7
Hs         TGTACTGATTTTGTGGCCAGA-TGCAATGATCTTTAAAAAC—AAATCTTTTCAATGGCA 1341
Pt         TGTACTGATTTTGTGGCCAGA-TGCAATGATCTTTAAAAAC—AAATCTTTTCAATGGCA 1349
Mmul       TGTACTGATTTTGTGGCCAGAATGCAATGATCTTTAAAAAC—A—TCTTTTCAGTGGCA 1344
Cj         TCTACTGATTTTGCAGCCACAATGCAATGATCTTCAGAAAC—AAATCTTTTCAGTGGCA 1383
Mm         TC—CTG-----G-AA-AGAATACAATAAA-TTTAAAAACGTAAGTCCTT—AAAAATA 1236
           *   ***     *     * * * **** *   ** * ****  *  ** **  *     *

                                                                  Mm PAS8
Hs         TAAGAAGTTGACAAAAATTTCTTAAAGTGCAATAGATTTTCAAGTGTATTGTGCCTTGTT 1401
Pt         TAAGAAGTTGACAAAAATT----AAAGTGCAATAGATTTTCAAGTGTATTGTGCCTTGTT 1405
Mmul       TAAGAAGTTGGCAAAAATTTCTTAAAGTGCAATAGATTTTCAAGTGTATTGTGCCTTGTT 1404
Cj         TCAGAAGTTGGCAAAA-TTTGTTAAAGTGCAAGAGATTTTCAAGTGTCTTGTGCCTTGTT 1442
Mm         CTATAAGTTGATAAATTTTTCTAAAAGTGCAATAGATTTTCAAGTGTATTGTGCCTTGTA 1296
             * ******  ***   **    ********* ************** ***********

Hs         CTAAAACTTTTAAGTAGGTGCACTTGACAGTATTG---AGGTCATTTGTTAAGGTGCTAT 1458
Pt         CTAAAACTTTTAAGTAGGTGCACTTGACAGTATTG---AGGTCATTTGTTAAGGTGCTAT 1462
Mmul       CTAAAACTTACAAGTAGGTGCACTTGACAGTATTG---AGGTCATTTGTTAAGGTGCTAT 1461
Cj         CTAAAATTTATAAGTAGGTGTACTTTACAGTATTG---AGGTCATTTGTTAAGGTGCTAT 1499
Mm         TTAAAATTTTAAAGTAGGTGTACTTGACAGTATTATCAAGGTCATTTGTTACGGTGCTAT 1356
            ***** **  ********* **** ********    ************* ********

Hs         TTCAATTAGTGTAGGTTTAGACTCTTGTACATTTCTCCCATAACTTTTTACAAAGTA--- 1515
Pt         TTCAATTAGTGTAGGTTTAGACTCTTGTACATTTCTCCCATAACTTTTTACAAAGTA--- 1519
Mmul       TTCAATTAGTATAGGTTTAGACTCTTGTACATTTCTCCCGTAACTTTTTACAAAGTACTT 1521
Cj         TTCAATTAGTATAAGTTTAGACTCTTGTACATTTTGCCCATAACTTTTTACAAAGCA--- 1556
Mm         TTCAATTA-TAGAAGTTTAGACT---GTACATT--GCCCATAACTTTTTACAAAGTACTT 1410
           ******** *  * *********    *******    *** *************** *

Hs         -TTTTGTTGCACATTCAGAGAATTTTATATATATA-----TGT-------CTTG---T-- 1557
Pt         -TTTTATTGCACATGCAGAGAATTATATATATATA-----TGT-------CTTG---T-- 1561
Mmul       ATTTTATTGCACATTCAGAGAATTTTATATATATA-----TGT-------CTTG---T-- 1564
Cj         --CTTACTGCAGATTGAGAGAATTTTATATATG-AC----TGT-------CTTG---T-- 1597
Mm         ATTTTATTGCACATGAAGAGAATTTTATATGTGTATCTTATGTACAAGTCCTTGAAGTTC 1470
              **  **** **  ******** ***** *  *      ***       ****   *
```

```
Hs              -G--TGGGTGTCCTCGAC--C--T-T-CCAAT--CTTATTTCG------TCTCT-TGGA- 1598
Pt              -G--TGGGTGTCCTCGAC--C--T-T-CCAAT--CTTATTTTG------TCTCT-TGGA- 1602
Mmul            -G--TGCGTGTCCTCGAC--C--T-T-CCAGT--CTTATTTTG------TTTCT-TGGA- 1605
Cj              -G--TGTGTGTTCTCAAT--C--T-C-CCAGT--TTTATTTCA------TCTCT-CAGA- 1638
Mm              CAATTGGATATTGTTGATTACAGTGTGTCAAGAACTTGGTTCAGAAAATTTCCATGGAC 1530
                    **  * *  *  *   *  *    **     **  **        * **    **

Hs              ----GA-T--T-GTT--GAA--------TGCAGCC---AGTG-----A-A-G-----AA- 1624
Pt              ----GA-T--T-GTT--GAA--------TGCAGCC---AGTG-----A-A-G-----AA- 1628
Mmul            ----GA-T--T-GTT--GAA--------TGCAGCC---AGTG-----A-A-G-----CA- 1631
Cj              ----GA-T--T-GTT--GAA--------TGCAGCC---AGTC-----A-A-G-----AA- 1664
Mm              CATAGAATGATAGTTAAGAAAAACTATTTGCAGCCTGAAGTGGTGGCACACGCCTTTAAT 1590
                    ** *  * ***  ***        *******   ***      * * *      *

Hs              -GTAG-ATTC------CT-A---A-----ATTT-T--ATTGGGGACCA---TG----GAA 1657
Pt              -GTAG-ATTC------CT-A---A-----ATTT-T--ATTGGGGACCA---TG----GTA 1661
Mmul            -GTAG-ATTC------TA-A---A-----ATTT-T--ATTGGGGACCA---TG----GAA 1664
Cj              -GTAG-ATTG------CA-A---A-----ATTT-T--ATTTGGGACCG---TG----GAA 1697
Mm              TGCAGCACTCAGGAGGCAGAGGCAGGTGGATTTCTGAATTTGTGGCCAGCCTGTTCTGCA 1650
                 * ** * *          *   *     **** *  *** * * **    **    * *

Hs              TGGT-AGTT----GA--G--A---------AGA-AAAC--TATTT--GCACAC-----AA 1689
Pt              TGGT-AGTT----GA--G--A---------AGA-AAAC--TATTT--GCACAC-----AA 1693
Mmul            TGGT-AGTT----GA--G--A---------AGA-AAAC--TATTT--GCACAC-----AA 1696
Cj              TGAT-AGTT----GA--G--A---------AGA-AAAC--TGTTT--GCACAC-----AA 1729
Mm              TAGTGAGTTCCAGGACAGCTAGGGCTCTACAGAGAAACCTTGTCTCAAAAAACCAAAAAA 1710
                *  * ****    **  *  *         *** ****  * * *    * **     **

Hs              CAGA---------------------------------T-TTT--AGATA-----CTTTTT 1708
Pt              CAGA---------------------------------T-TTT--AGATA-----CTTTTT 1712
Mmul            CAGA---------------------------------T-TTT--AGATA-----CTTTTT 1715
Cj              CAGA---------------------------------T-TTT--AGATA-----CTTTT- 1747
Mm              AAGAAAAAAAAGAAAAAGAAAAAAAAGAAAGAAAAACTATTTGCACATAACAGACTTTTT 1770
                 ***                                 * ***  * ***     *****

Hs              GCTGCTAG-TTGTGTAATATTTATTGAACATTTTGACAAATATTTATTTTTGTAAGCCTA 1767
Pt              GCTGCTAG-TTGTGTAATATTTATTGAACATTTTGACAAATATTTATTTTTGTAAGCCTA 1771
Mmul            GCTGCTAG-TTGTGTAATATTTATTGAAAATTTTGACAAATATTTATTTTTGTAAGCCTA 1774
Cj              GCTGCTAG-TTGTATAATATTTATTGAAAATTTTGACAAATATTTATTTTTGTAAGCCTA 1806
Mm              GCTGCTAGGTTGTGTAATATTTATTGAACATTTTGACAAATATTTATTTTTGTAAGCCTA 1830
                ******** **** ************** *******************************

                                                             Mm              PAS9
Hs              AAAATGATTCTTTGAAAGTTTAAAGAAACTTGACCAAAAGACAGTAC--AAAAA-ACACT 1824
Pt              AAAATGATTCTTTGAAAGTTTAAAGAAACTTGACCAAAAGGCAGTAC--AAAAA-ACACT 1828
Mmul            AAAATGATTCTTTGAAAGTTTAAAGAAACTTGACCAAAAGACAGTAC--AAAAACACACT 1832
Cj              AAAATGATTCTCTGAAAGTTTAAAGAAACTTGACCAAAAGACAGTAC--AGAAA-ACACT 1863
Mm              AAAATGATTCTTTGAAAGCTTAAAGAAACTTGACCAAAAGACAGTATTAAAAAAAACACT 1890
                *********** ****** ********************* *****   * *** *****

Hs              GGCACTTGAATGTTGAATGTCACCGTATGTGAAATAATATATTTGGGGTAGTGTGAGCT 1884
Pt              GGCACTTGAATGTTGAATGTCACTGTATGTGAAATAATATATTTCGGGGTAGTGTGAGCT 1888
Mmul            GGCACTTGAATGTTGAATGTCACCGTATGTGAAATAATATATTTCGGGGTAGTGTGAGCT 1892
Cj              GGCACTTGAATGT--A-----ACCGTATGTGAAATAATATATTTTCTGGTAGTGTGCGCT 1916
Mm              GGTATTTGATTGT-------CACTGTATGTGAAATAGTATATTTTG-GGTAATGTGAGCT 1942
                ** * **** ***        ** ************ *******   **** **** ***

                               Mac + Cj                                      PAS4
Hs              TTT-AATGTTAAGTC-TGTTAAACTTGAGTCAAATTAAGCAGACCCGGCATTGGCAATGT 1942
Pt              TTT-AATGTTAACTC-TGTTAAACTTGAGTCAAATTAAGCAGACCCGGCATTGGCAATGT 1946
Mmul            TTT-AATGTTAAGTCCTATTAAACTTGAGTCAAATTAAGCAGACCTGGCATTGGCAATGT 1951
Cj              TTT-AATGTCAAGTCCTATTAAACTTGAGTCAAATTAAGCAGACCCGGCATTGGCAATGT 1975
Mm              TTTTAATGTTAAGTTCTACTAAACTTGAGTCAAATTGATCTAACCCAGTATTAGTGATGT 2002
                *** ***** ** *  *  **************** * *  ***  * *** *  ****

Hs              AGCTGTA--ATTTT-CT-----------GACAAAATTTAAGACAAAATTGTCAACT-TGA 1987
Pt              AGCTGTA--ATTTT-CT-----------GACAGAATTTAAGACAAAATTGTCAACT-TGA 1991
Mmul            AGCTGTA--ATTTT-CT-----------GACAAAATTTAAGACAAAATTGTCAACT-TGA 1996
Cj              AGCTATA--ATTTT-CT-----------GAGAAAATTTAAGACAAAATTGTCAACT-TGA 2020
Mm              AGCTACACTATTTTTCTTTTTTTCTTTTGACAAAATGAAAGGCAA--TTGG-AATAATGA 2059
                ****  *  ***** **           ** * ***  *** ***  ***  **   ***
```

```
                Cj                                                           PAS5
Hs              AA--CTAAAACATGCCAAGGTTTTGATATACTTGTCTTAAGATATTAATGAAACACTTCT 2045
Pt              AA--CTAAAACATGCCAA-----TGATATGCTTGTCTTAAGATATTAATGAAACACTTCT 2044
Mmul            AA--CTAAAACATGCCAAGGTTTTGATATACTTGTCTTAAGATATTAATGAAACACTTCT 2054
Cj              A---TTAAAACTTGCCACGGTTTTGATATCCTTGTCTTATGGTATTAATGAAACTCATCT 2077
Mm              AAATTTAAAACATGCCAAGGTTTTGATG----TAT-TTAAGATATTAGTGAAA-AC---T 2110
                *    ****** *****      ****     * * *** * ***** *****  *   *

Hs              GAACACTGATAGGAAGTGTCCACATCCACAA-A-GTTTCTCTTGAGTTTTGTTATGTGTT 2103
Pt              GAACACTGATAGGAAGTGTCCACATCCACAA-A-GTTTCTCTTGAGTTTTGTTATGTGTT 2102
Mmul            GAATGCTGATAGGAAGTGTCCACATCCACAACA-GTTT-TCT-GAGTTTTGTTATGTGTT 2111
Cj              GAACACTGATATGAAGTGTCTACATC-GTAG-ATGCTC-TCT-GAGTTT-GTTAGATGTT 2132
Mm              AAAAATT--TTCTCAGT-TT---GTT--TGA-A--TTTACCT----TAC--TTACATGAT 2153
                 **   *  *    *** *     *       *   *   **    *    ***  ** *

Hs              TTGTTGTGTTT-----GATTTTCAGTGATTGTCTGGTATATTTACAGTCCTCAAAC---A 2155
Pt              TTGTTTTGTTT-----GATTTTCAGTGATTGTCTGGTATATTTACAGTCCTCAAAC---G 2154
Mmul            TTGTTTTGTTTTGTTTGATCTTTGGTGATTGTCTGGCATATTTACAGTCCTCAAACAT-G 2170
Cj              TTGTTTTG--------AAT-TTCAGTGATTGTCTGGCATATCTACAGTCCTCAAACGTTA 2183
Mm              CTATT-TGTTC-----AGTATT--GTAACTGTCTGGCATATTTATAATCTTCAAACAT-G 2204
                 * ** **          * **  ** * ******* **** ** * ** ******

Hs              TG-----GT-----TATTTCTGT---CAGTGAC---TTAACATTCGGTTTTATCAGCCAG 2199
Pt              TG-----GT-----TATTTCTGT---CAGTGAC---TTAACATTCGGTTTTATCAGCCAG 2198
Mmul            TG-----GT-----TATTTTT-T---CAGTGAC---TTA-CATTCAGTTTTATCAGCCAG 2212
Cj              TATTTTTGT----GTATTGCCGTGAGCAGTGAC---TTA-CATTCAGTTTCATCAGCCAG 2235
Mm              TGGTTATATCTTTGTATTGCCATAATCAGTGATGTTTTA-CATTCAGTTT-ATCAGCCAG 2262
                *       *     ****    *   ******    *** ***** **** *********

                              Hs + Pt                                        PAS4
Hs              CAGTATTCTTCAGTAAATAAAGA-ATGGAATT-GCTGAATGTAATCATTGAACCTCGAGT 2257
Pt              CAGTATTCTTCAGTAAATAAAGA-ATGGAATT-GCTGAATGTAATCATTGAACCTCGAGT 2256
Mmul            CAGTATT-TTCAGTA----AAGA-ATGGAATT-GCTGAATGTAACCGTTGAACCTCGAGT 2265
Cj              CAATATT-TTCAGTA----AAGA-ATGTAATT-GCTGAATGTAACCATTGACCCTCAAGT 2288
Mm              CAATACT-TTCAGTAAAT-AATACACTAAATTTGCTGACTGTAGACATGGAACTTTAAGT 2320
                ** ** * *******    ** * *   **** ***** ****  * * ** * *  ***

Hs              CACTGTAAAAGTTCAGTAATTGCTT-ATTGTATTAGTTTTAGATGCTGGCACTGCATGTG 2316
Pt              CACTGTAAAAGTTTAGTAATTGCTT-ATTGTATTAGTTTTAGATGCTGGCACTGCATGTC 2315
Mmul            CACTGTAAAATTTTAGTAATTGCTT-ATTGTATTAGTTTTAGATGCTGGCACTGCATGTG 2324
Cj              CACTCTA--AGTTTAGTAATTGCTT-ATTGTATTAGTTGCAGATGCTGTTACTGCATGTG 2345
Mm              TTCTATAA-AGTT-AGTAATTGCTTTATTGTATTAGTATTAGGTGCTAGCACTGCATATG 2378
                  ** **  * ** *********** ***********   ** ****   ******* *

                                        Hs + Pt + Mac PAS5/ Cj PAS6/ Mm PAS10-11
Hs              CTCTG-TTTATTCTGATTTTACTAAAATAAAAAGT-TCA-AAAGTCT-TCCT--GCTGTC 2370
Pt              CTCTG-TTTATTCTGATTTTACTGAAATAAAAAGT-TCA-AAAGTCT-TTCT--GCTGTC 2369
Mmul            CTCTG-TTTATTCTGATTTTACTAAAATAAAAAGT-TCA-AAAATCT-TTTTT-GCTGTC 2379
Cj              CTCTG-TT-ATTCTGATTTTACTACAATAAAAAGT-T-A-AAAATCTGTTTTCTGCTGTC 2400
Mm              -TCTGATCTATTCTCATTTTATTAAAATAAAAAGTATAGCAAAATGT-TT----ACTGTA 2432
                 **** *  ***** ****** *  ********** *   *** * * *      ****

Hs              ACTTGTAGCTTAATGTTGAGTTGAAGATGAACTTGTGCTCT------------------- 2411
Pt              ACTTGTAGCTTAATGTTGAGTTGAAGATGAACTTGTGCTCTTAACCTGAAGATTGGAAAT 2429
Mmul            ACTTGTAGCTTAATGTTGAGTTGAAGATGAATTTGTGCTCCTAACCTGAAGAT------- 2432
Cj              ACCAGTAGCTTAATGTTGAGTTGAAAATGAACTTGT---CTTAACCTGAAGATTGGAAAT 2457
Mm              ACTTGTGACTTAATGTTC--TTG----TGAACTTGCATGCTTGAACTG----TTGGAAAT 2482
                **  **  *********   ***    **** ***    *

Hs              -----------------------------------------
Pt              CTGATTTCTTGGCTAACTACCAAAGCTGTTTGTGAAATACT 2470
Mmul            -----------------------------------------
Cj              CTGGTATCTTGGCTAACTAC--------------------- 2477
Mm              CT--------------------------------------- 2484
```

Figure S2 Vogt et al.

Schematic view on locations of the distinct *DDX3Y* testis transcription start sites in human exon T (T-TSS-I; T-TSS-II) and exon1 (TSS-I(T)) according to Rauschendorf et al.[22] . Additional start sites „TSS-I-*ext*“ and „TSS-I“ marks the range of the 5´UTR lengths found in somatic *DDX3Y* transcripts in human and NHP.

Putative *DDX3Y* tanscripts polyadenylation sites PAS1-5 mapped *„in silico“* (see Fig. 1S) are marked with striped triangles in the *DDX3Y* 3´UTR downstream of the TGA translation stop codon in exon 17. The *„outer primers*“ forward given here below exon T and exon 1 (stippled lines) are combined with the *„outer primers*“ reverse given below the 3´UTR upstream of each putative PAS (stippled lines) in the first round of the nested RT-PCR assay. By this approach, all *DDX3Y* transcript variants expressed in testis, kidney and liver tissue, are amplified, separately. Their number code and sequence positions in the genomic human BAC clone given at the right are listed in Table S1, together with all primer data of the corresponding NHP RT-PCR assays.

.

DDX3Y testis transcript start sites in distal (T-TSS-I/II) and proximal promoter (TSS-I(T))

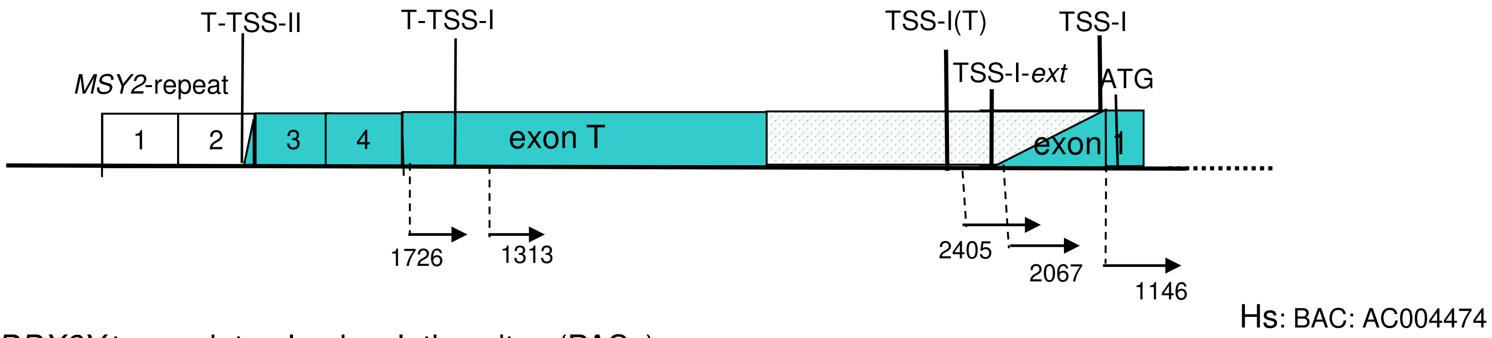

DDX3Y transcript polyadenylation sites (PASs)

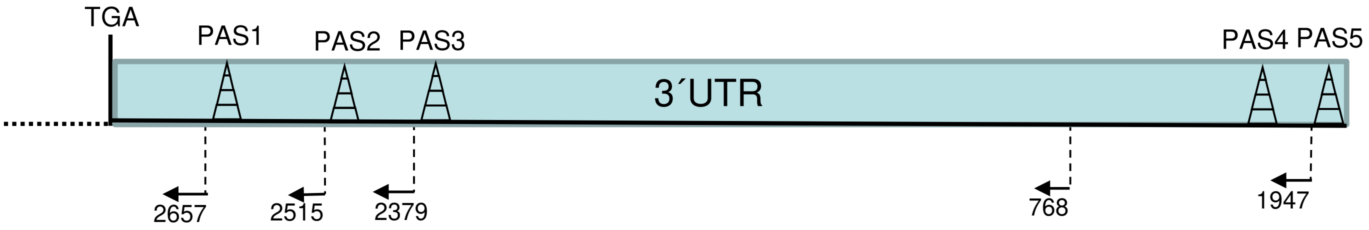

Figure S3 Vogt et al.

Schematic view on locations of the distinct *DDX3Y* transcripts polyadenylation sites PAS1-5 (+6 in Cj). They are marked with striped triangles in the *DDX3Y* 3´UTR downstream of the TGA translation stop codon. PAS related locations of „*outer*“ primers used for the first round of all nested PCR assays are marked with stipple lines (see also Fig. 2S).
A common forward primer is used for the second PCR round. It is bridiging *DDX3Y* exon 16-17. PCR amplification products produced together with the reverse PAS related „*inner*“ primers (see lines mark) then reveal which PAS´s is used for any *DDX3Y* transcripts expressed in human (Hs) and each primate tissue analysed: Pt: Pan trogloydytes; Mmul: Macaca mulatta; Cj: Callithrix jacchus. Their number code and sequence positions in the genomic BAC clones given at the right are listed in Table S1.
.

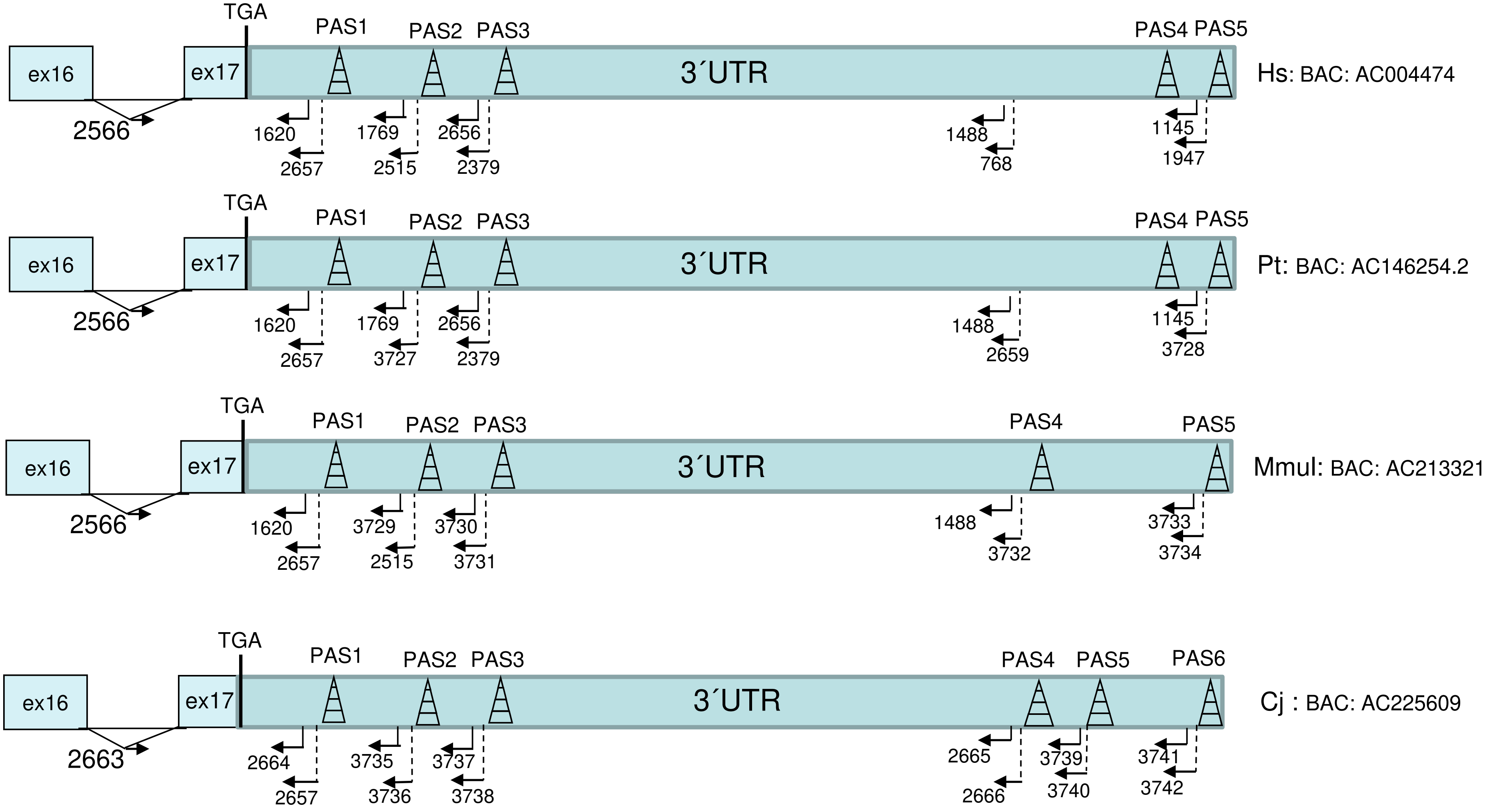

Supplementary informations for Vogt et al. 2022: Figure S4

originals of Westernblots in Fig. 3 A+B and description of their processing (cropping) for mproving clarity and conciseness of Figure 3

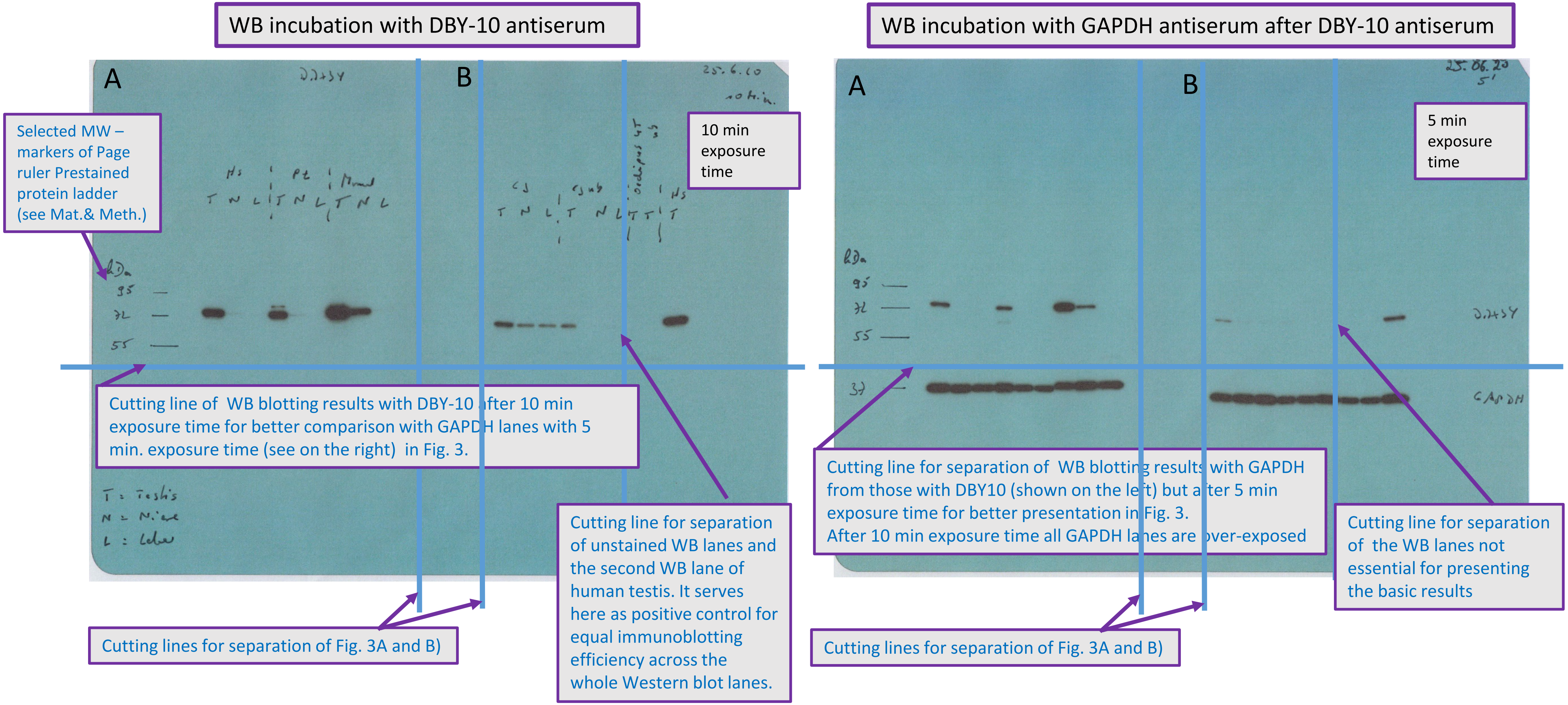

Figure S5

Double-Immuno-Fluorecence (DIF) staining pattern of DDX3Y (**A**: green) and Ki67 (**B**: red) marks overlap of expression in germ cells of testicular tissue sections of Callithrix jacchus newborn in merged picture: **C**.

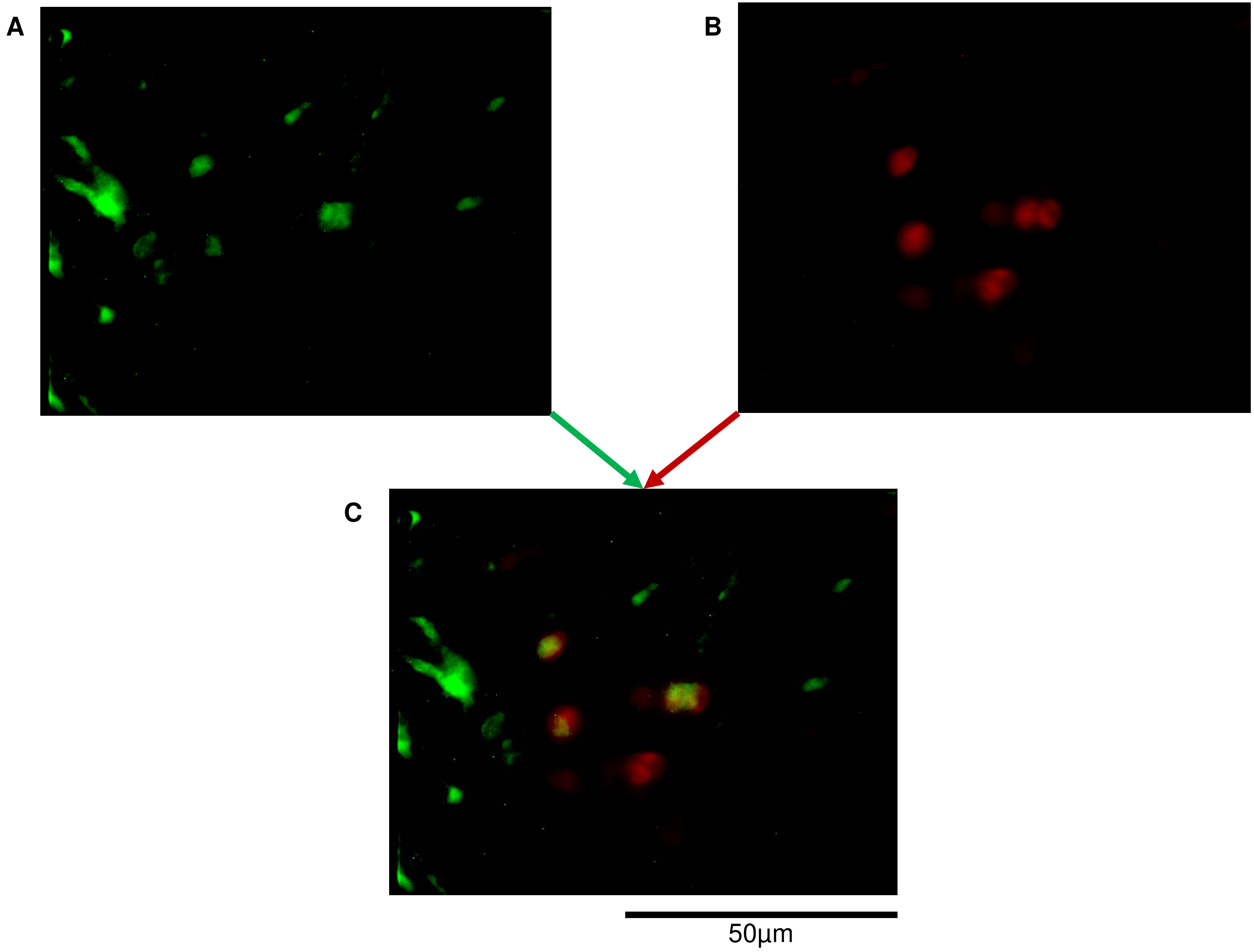

Table S1**:** List of "*outer*" and "*inner*" forward and reverse prime**r** pairs used for identification of 5´UTR-3´UTR long *DDX3Y* transcript variants with nested RT-PCR assays. Human and each Non-human primate (NHP) primer set is listed separately. Each *DDX3Y* transcript variant starting at one of the transcriptional start sites "TSS" , respectively, is polyadenylated after one of the "PASs" given at the left, can be amplified separately by this work flow for each tissue in each species analysed. See also supplementary Fig. S2 and S3. Lab code of each primer used and associated BAC sequence start position (5´ to 3´) is given in the third and forth table column; sequences are displayed at the right.

Human *DDX3Y* 5´UTR - 3´UTR transcript variants

Transcriptional start sites (TSSs)	Nested RT-PCR assay: *work flow*	Lab code primer no.	BAC 475I1 (AC004474) start sequence position	primer sequence 5´ to 3´
T-TSS-II	*First nested RT PCR round : outer primers forward*	1726	54263	CTGTGATGCTAAAGCCGTATGC
T-TSS-I		1313	54362	TCAAGTCTGTCGAGCCTCTG
TSS-I(T)		2405	54925	TAGGGAGAAGTAACGGTAGC
TSS-I-*ext*		2067	55031	CATATTACCGCGTAGGCTAA
TSS-I		1146	55087	TTCCGCTATTCGGTCTCACAC
Polyadenylation sites (PASs)				
PAS1	*First nested RT-PCR round: selection for 3´UTR polyadenylation sites (PASs) with outer primers reverse*	2657	68478	GTGACTTTGAGCTCCCTTGAA
PAS2		2515	68715	GTCAAAACAATTGGTATCGTGAG
PAS3		2379	68884	GATGTTCTCCCAACCCCAAGTC
PAS4		768	69321	CTGTTTAAACCGTTCTTGACC
PAS5		1947	70600	GAACTTTTACAGTGACTCGAG
DDX3Y exon16-17	common *inner primer forward*	2566	67741	GGAGGTGGCTATGGAGGCTTC
PAS1	*Second nested RT-PCR round: identification of last PAS site used with PAS specific set of inner primers reverse*	1620	68442	CAGCTGGTAATGTACTGCAAG
PAS2		1769	68683	GCCAACAGGACATCAAAGTAG
PAS3		2656	68770	GTATCATACCTCTGTGCCAAG
PAS4		1488	69273	AAGACCAGAATCCAACCAGGC
PAS5		1145	70576	CAATGATTACATTCAGCAATTCC

Pan troglodytes *DDX3Y* 5´UTR - 3´UTR transcript variants

Transcriptional start sites (TSSs)	Nested RT-PCR assay: *work flow*	Lab code primer-no.	BAC CH251-128L22 (AC146254.2) start sequence position	primer sequence 5´ to 3´UTR
T-TSS-II	*First nested RT PCR round : outer primers forward*	1726	59906	CTGTGATGCTAAAGCCGTATGC
T-TSS-I		2422	59807	TCAAGCCTGTCGAGCCTCTG
TSS-I(T)		2405	59244	TAGGGAGAAGTAACGGTAGC
TSS-I-*ext*		2420	59139	CATATTACCGCGTAGGTTAAGC
TSS-I		1146	59083	TTCCGCTATTCGGTCTCACAC
Polyadenylation sites (PASs)				
PAS1	*First nested RT-PCR round: selection for 3´UTR polyadenylation sites (PASs) with outer primers reverse*	2657	45634	GTGACTTTGAGCTCCCTTGAA
PAS2		3727	45396	GTCAAAACAATTGGTATCATGAG
PAS3		2379	45226	GATGTTCTCCCAACCCCAAGTC
PAS4		2659	44786	CTGTTTAAACCGTTCTTGACC
PAS5		3728	43516	AAACTTTTACAGTGACTCGAG
DDX3Y exon16-17	common *inner primer forward*	2566	46365	GGAGGTGGCTATGGAGGCTTC
PAS1	*Second nested RT-PCR round: identification of last PAS site used with PAS specific set of inner primers reverse*	1620	45670	CAGCTGGTAATGTACTGCAAG
PAS2		1769	45428	GCCAACAGGACATCAAAGTAG
PAS3		2656	45340	GTATCATACCTCTGTGCCAAG
PAS4		1488	44834	AAGACCAGAATCCAACCAGGC
PAS5		1145	43540	CAATGATTACATTCAGCAATTCC

Macaca mulatta *DDX3Y* 5´UTR - 3´UTR transcript variants				
Transcriptional start sites (TSSs)	Nested RT-PCR assay: *work flow*	Lab code primer no.	BAC CH250-541A11 (AC213321) start sequence position	primer sequence 5´to 3´
T-TSS-II	*First nested RT PCR round : outer primers forward*	1726	83880	CTGTGATGCTAAAGCCGTATGC
T-TSS-I		2530	83781	TCAAGCCTGTCGAGCTTGTG
TSS-I(T)		2529	83212	TAGGCAGAAGTAATGATAGC
TSS-I-ext		2067	83107	CATATTACCGCGTAGGCTAA
TSS-I		2660	83050	TCAGCTACTCGGTCTCGCAC
Polyadenylation sites (PASs)				
PAS1	*First nested RT-PCR round: selection for 3´UTR polyadenylation sites(PASs) with outer primers reverse*	2657	69448	GTGACTTTGAGCTCCCTTGAA
PAS2		2515	69211	GTCAAAACAATTGGTATCGTGAG
PAS3		3731	69042	GTTCTCCCAACCCCCAAGTC
PAS4		3732	68600	CTGTTTAAACCCTTCTTGACC
PAS5		3734	67314	TACTAAAATTTTACAGTGACTCGAG
DDX3Y exon16-17	common *inner primer forward*	2566	70188	GGAGGTGGCTATGGAGGCTTC
PAS1	*Second nested RT-PCR round: identification of last PAS site used with PAS specific set of inner primers reverse*	1620	69485	CAGCTGGTAATGTACTGCAAG
PAS2		3729	69241	GAGCCAAAAGGACATCAAAG
PAS3		3730	69154	GTATCATACCTGTGTGCCAAG
PAS4		1488	68648	AAGACCAGAATCCAACCAGGC
PAS5		3733	67342	CAACGGTTACATTCAGCAATTCC
Callithrix jacchus *DDX3Y* 5´UTR - 3´UTR transcript variants				
	Nested RT-PCR assay: *work flow*	Lab code primer- no.	BAC CH259-161K20 (AC225609) start sequence position	primer sequence 5´to 3`
T-TSS-II	First nested RT PCR round : outer primers *forward*	2532	52459	CTGTGACGCTAAGGTCGTATGC
T-TSS-I		2533	52360	TCAAGCCCGTAAAGTATCTGG
TSS-I(T)		2381	51850	TAGGCGCAAGGAAACGTACC
TSS-I-*ext*		2319	51749	ACCATTTTAAGACGGAGTCTAAG
TSS-I		2662	51707	CTCCGATATTCGGTCTCACC
Polyadenylation sites (PASs)				
PAS1	*First nested RT-PCR round: selection for 3´UTR polyadenylation sites (PASs) with outer primers reverse*	2657	39132	GTGACTTTGAGCTCCCTTGAA
PAS2		3736	38893	GTCAAAACAGTGGGTATCGTGA
PAS3		3738	38427	CGCCAAGACTGCAAGAGTC
PAS4		2666	38246	CTGTTTAATTTCCTTCTTGACTG
PAS5		3740	37293	CTGAAAATTATAGCTACATTGCC
PAS6		3742	36979	CTAAACTTAGAGTGACTTGAG
DDX3Y exon16-17	common *inner primer forward*	2663	39583	AGAGGTGGCTATGGAGGCTTC
PAS1	*Second nested RT-PCR round: identification of last PAS site used with PAS specific set of inner primers reverse*	2664	39168	CTGCTGGTAACGTCCTGCAAG
PAS2		3735	38924	ATCCAACAGGACATCAAAGTAG
PAS3		3737	38837	GTATCACACCTGTGTGCCAC
PAS4		2665	38296	AAGACCAGAAACCAATCAGGC
PAS5		3739	37319	CGGGTCTGCTTAATTTGACTC
PAS6		3741	37003	CAATGGTTACATTCAGCAATTAC

Table S2 Vogt et al.

Intensities of PCR amplification products from all *DDX3Y* transcript variants after second round of the nested RT-PCR assay in human and primates. The putative major Transcriptional Start Site (TSS) with the putative major PolyAdenylation Site (PAS) in testis tissue is marked with larger **bold** crosses"**+**". Detailed information for all TSSs and PASs listed here are given in Table S1 and the Materials & Methods section.

DDX3Y transcripts	H. sapiens			P. troglodytes			M. mulatta			C. jacchus			3´UTR polyadeny-lation
TSS	testis	kidney	liver	testis	kidney	liver	testis	kidney	liver	testis	kidney	liver	PAS
T-TSS-II	+w	-	-	+w	-	-	+w	-	-	-	-	-	PAS1
T-TSS-I	**+**	-	-	**+**	-	-	+	-	-	-	-	-	
TSS-I(T)	+w	-	-	+w	-	-	**+**	-	-	**+**	+w	-	
TSS-I-*ext*	+	+	+	+	+	+	+	-	-	+	+	+	
TSS-I	+	+	+	+	+	+	+	+	+	+	+	+	
T-TSS-II	+w	-	-	-	-	-	+w	-	-	-	-	-	PAS2
T-TSS-I	**+**	-	-	**+**	-	-	+	-	-	**+**	-	-	
TSS-I(T)	+	-	-	+w	-	-	**+**	-	-	**+**	-	+w	
TSS-I-*ext*	+	+	+	+	+	+	+w	-	-	+	-	+	
TSS-I	+	+	+	+	+	+	+	+	+	+	+	+	
T-TSS-II	-	-	-	-	-	-	-	-	-	+w	-	-	PAS3
T-TSS-I	-	-	-	-	-	-	-	-	-	+	-	-	
TSS-I(T)	-	-	-	-	-	-	-	-	-	**+**	-	+w	
TSS-I-*ext*	-	+w	+w	-	+	+	-	-	-	+	+	+	
TSS-I	-	+	+	-	+	+	+	+	+	+	+	+	
T-TSS-II	-	-	-	-	-	-	-	-	-	-	-	-	PAS4
T-TSS-I	-	-	-	-	-	-	-	-	-	**+**	-	-	
TSS-I(T)	-	-	-	-	-	-	-	-	-	+	-	-	
TSS-I-*ext*	-	+w	+w	-	+	+	-	-	-	+	+	+	
TSS-I	-	+	+	-	+	+	-	+	+	+	+	+	
T-TSS-II	-	-	-	-	-	-	-	-	-	-	-	-	PAS5 =PAS6 in *Callithrix jacchus*
T-TSS-I	-	-	-	-	-	-	-	-	-	-	-	-	
TSS-I(T)	-	-	-	-	-	-	-	-	-	-	-	-	
TSS-I-*ext*	-	+	+	-	-	-	-	-	-	**+**	+	+	
TSS-I	-	+	+	-	-	-	-	+w	+w	+	+	+	

"+", visible PCR amplification product; "+w", weak visible PCR amplification product; "**+**", major PCR amplification product in testis, i.e. : major PAS site used for *DDX3Y* transcripts with the associated PAS site; "-", no PCR amplification product visible.

Table S3: Sequences of designed 6FAM marked TaqMan probes and flanking oligonucleotides used in TaqMan assays for specific amplification of all *DDX3Y* transcripts polyadenylated at PAS1 or PAS2 in proximal 3´UTR, respectively, in distal 3´UTR (PAS4-6) of Hs (Human sapiens), Ptro (Pan troglodytes), Mmul (Macaca mulatta) and Caja (Callithrix jacchus).

DDX3Y 3´UTR sequence upstream of:	species	TaqMan Probe (5´- 3´)	F(orward) and R(reverse) primer sequences (5´- 3´)	GenBank accession no. And polarity of BAC sequence used for primer design	BAC sequence start and end position	Sizes (nt) of amplification products
PAS1	Hs	(6FAM) TACAAAGAAGCTAATATGGAAAC	F-AACTGAATCTGCTTTGCAGCAA	AC004474.1	69327 - 68465	138
			R-CCCTTGAATTATCAGGAGAATCACA			
	Ptro	(6FAM) CCTTTAAAGAAGCTAATATGGAA	F-AACTGAATCTGCTTTGCAGCAA	AC146254.2 (minus strand)	45788 - 45647	142
			R-CCCTTGAATTATCAGGAGAATCACA			
	Mmul	(6FAM) TACAAAcAAGCTAATATGGAAAC	F-AACTGAATCTGCTTTGCAGCAA	AC213321.3 (minus strand)	69599 - 69461	139
			R-CCCTTGAATTATCAGGAGAATCATA			
	Caja	(6FAM) TACAAAcAAGCTAATATGGAAAC	F-AACTGAATCTGCTTCGCAGCAA	AC225609.4 (minus strand)	39283 - 39145	139
			R-CCCTTGAATTTTCAGGACAATCACT			
PAS2	Hs	(6FAM) TGTGACTGAGGATCGTTT	F-CTCTTCCCCTCCTGCTTTAGTG	AC004474.1	68559 - 68686	128
			R-TGAGCCAACAGGACATCAAAGTAGT			
	Ptro	(6FAM) TGTGACTGAGGATCGTTT	F-CTCTTCCCCTCCTGCTTTAGTG	AC146254.2 (minus strand)	45553 - 45425	129
			R-TGAGCCAACAGGACATCAAAGTAGT			
	Mmul	(6FAM) TGTGACTGAGGATCGTTC	F-CTCTTCCCCTCCTGCTTTATTC	AC213321.3 (minus strand)	69367 - 69244	124
			R-TGATCCAAAAGGACATCAAAGTAAT			
	Caja	(6FAM) TGTGGCTAAGGATCGTTC	F-CTCTCCCCTCCTGCTTTAGTC	AC225609.4 (minus strand)	39049 - 38922	128
			R-TGATCCAACAGGACATCAAAGTGG			
PAS4-6*	Hs	(6FAM) CTACAGGCCTGGTTGGA	F-GACCATGGCAGTGACCAGG	AC004474.1	69223 - 69332	110
			R-CACAATCCTTCCTGTTTAAACCGT			
	Ptro	(6FAM) CTACAGGCCTGGTTGGA	F-GACCATGGCAGTGACCAGG	AC146254.2 (minus strand)	44884 - 44775	110
			R-CATAATCCTTCCTGTTTAAACCAT			
	Mmul	(6FAM) CTATAGGCCTGGTTGGA	F-GACCATGGCAGTGACCAGG	AC213321.3 (minus strand)	68698 - 68589	110
			R-CATAATCCTTCCTGTTTAAACCCT			
	Caja	(6FAM) CGGCAGGCCTGATTGGT	F-GGCCATGGCAACGACCAAG	AC225609.4 (minus strand)	38346 - 38235	112
			R-CATAATCCTTCCTGTTTAATTTCCT			

* This TaqMan probe has been designated as "PAS4-6" because located upstream of PAS4; it will thus amplify all *DDX3Y* transcripts polyadenylated downstream of PAS4 in the distal 3´UTR in each primate and not distinguish polyadenylation at PAS4 or PAS5 (PAS6 in Callithrix jacchus only), accordingly.

Table S4: Concentration of primers and TaqMan probes used in quantitative RT-PCR reactions displayed in Fig. 2

DDX3Y transcription polyadenylated at:	conc. primers (nM)		conc. TaqMan probe (nM)
	Forward	Reverse	
PAS1	1200	1200	400
PAS2	600	600	200
PAS4-6	900	900	250
control genes	6000 pM: probe and primer mix (µl per well)		
HPRT	2.75		
ACTB			

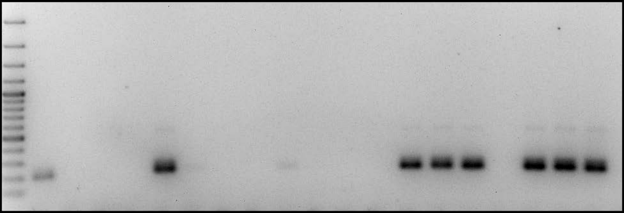

User1 Exp. Time: 2.00 sec Gain: 23 % Upper: 100 % Lower: 5 % Lin.Gamma: 7
Date: 29.11.2021 Time: 09:32:43

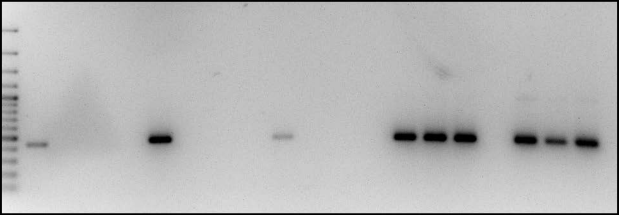

User1 Exp. Time: 2.00 sec Gain: 23 % Upper: 100 % Lower: 5 % Lin.Gamma: 7
Date: 29.11.2021 Time: 09:32:43

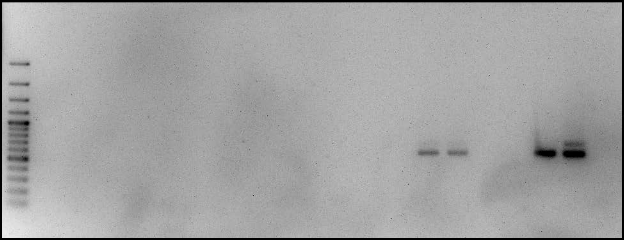

User1 Exp. Time: 2.00 sec Gain: 23 % Upper: 100 % Lower: 5 % Lin.Gamma: 7
Date: 29.11.2021 Time: 09:32:43

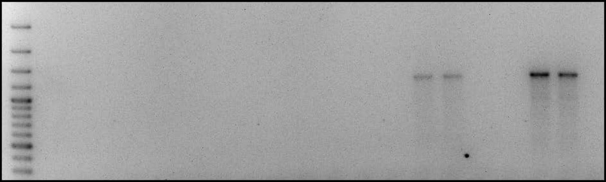

User1 Exp. Time: 2.00 sec Gain: 23 % Upper: 100 % Lower: 5 % Lin.Gamma: 7
Date: 29.11.2021 Time: 09:32:43

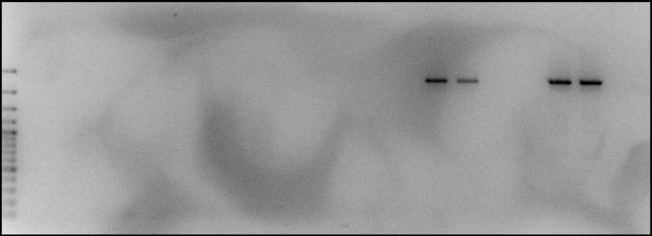

User1 Exp. Time: 2.00 sec Gain: 23 % Upper: 100 % Lower: 5 % Lin.Gamma: 7
Date: 29.11.2021 Time: 09:32:43

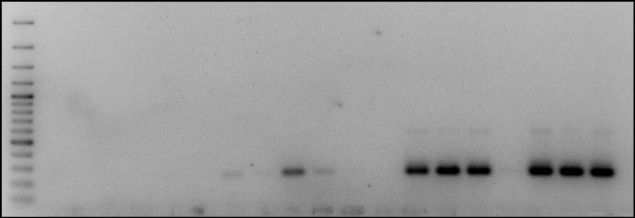

User1 Exp. Time: 2.00 sec Gain: 23 % Upper: 100 % Lower: 5 % Lin.Gamma: 0
Date: 29.11.2021 Time: 09:22:25

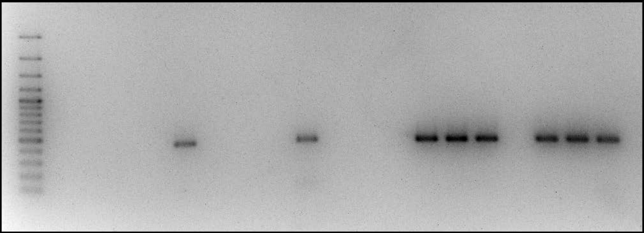

User1 Exp. Time: 2.00 sec Gain: 23 % Upper: 100 % Lower: 5 % Lin.Gamma: 0
Date: 29.11.2021 Time: 09:22:25

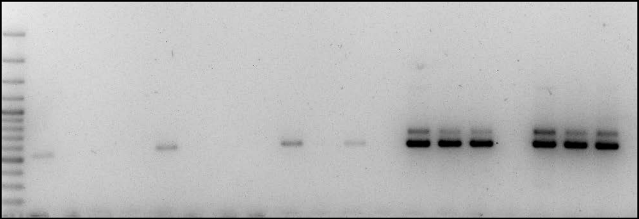

User1 Exp. Time: 2.00 sec Gain: 23 % Upper: 100 % Lower: 5 % Lin.Gamma: 0
Date: 29.11.2021 Time: 09:22:25

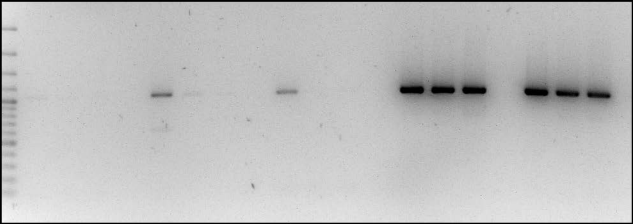

User1 Exp. Time: 2.00 sec Gain: 23 % Upper: 100 % Lower: 5 % Lin.Gamma: 0
Date: 29.11.2021 Time: 09:22:25

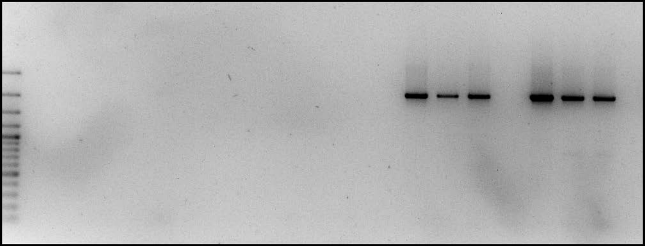

User1 Exp. Time: 2.00 sec Gain: 23 % Upper: 100 % Lower: 5 % Lin.Gamma: 0
Date: 29.11.2021 Time: 09:22:25

Quantitative Expression von DDX3Y-3´UTR-Transkripten in Humangewebe bezogen auf DDX3Y PAS1

25.06.2020

				PAS1	PAS2	PAS3
				Ct	Ct	Ct
Testis B				23,698	24,854	28,813
Niere ♂				26,432	26,452	26,425
Leber ♂				26,785	26,699	26,810

ΔCt control - sample				
		PAS1	PAS2	PAS3
		ΔCt	ΔCt	ΔCt
Testis B		0,000	-1,156	-5,115
Niere ♂		0,000	-0,020	0,007
Leber ♂		0,000	0,086	-0,025

Auswertung = [2 target^ (ΔCt control - sample)] / [2 reference^ (ΔCt control - sample)]

	Testis B	Niere ♂	Leber ♂			Testis B	Niere ♂	Leber ♂
	ΔΔCt	ΔΔCt	ΔΔCt			+ SD	+ SD	+ SD
**PAS1**	1,000	1,000	1,000		**PAS1**	0,089	0,065	0,065
**PAS2**	0,449	0,986	1,061		**PAS2**	0,102	0,085	0,078
**PAS3**	0,029	1,005	0,983		**PAS3**	0,085	0,098	0,098

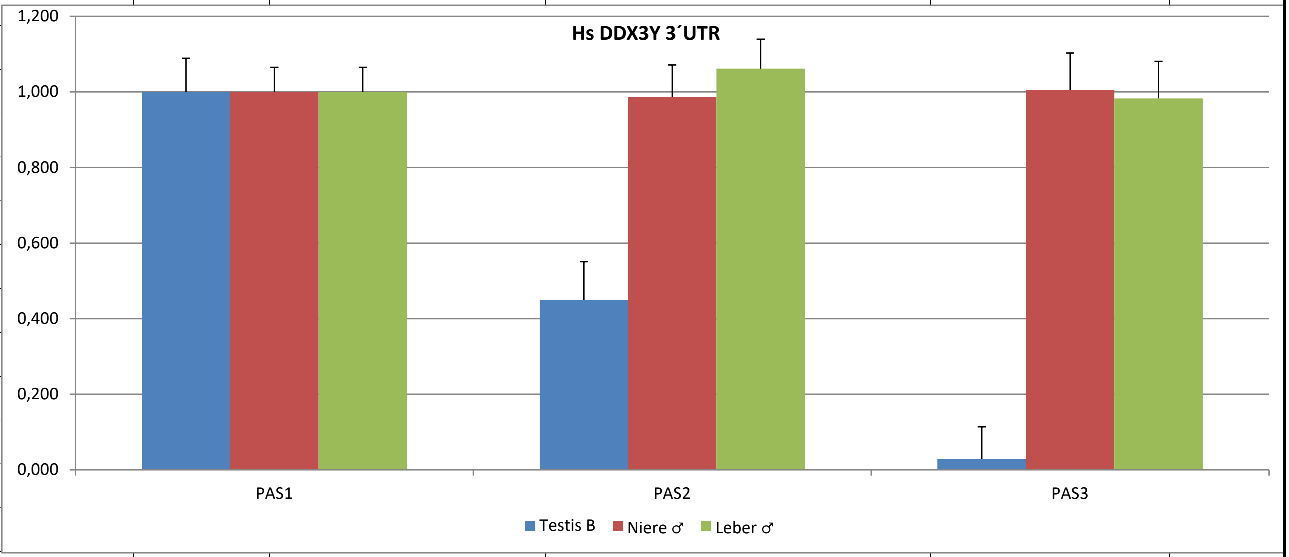

Quantitative Expression von DDX3Y-3´UTR-Transkripten in Gewebe von Pt bezogen auf DDX3Y PAS1

25.06.2020

	PAS1	PAS2	PAS3
	Ct	Ct	Ct
Testis	21,666	22,780	25,254
Niere ♂	22,760	22,750	22,747
Leber ♂	23,505	23,509	23,533

∆Ct control - sample			
	PAS1	PAS2	PAS3
	∆Ct	∆Ct	∆Ct
Testis	0,000	-1,114	-3,588
Niere ♂	0,000	0,010	0,014
Leber ♂	0,000	-0,004	-0,028

Auswertung = [2 target^ (∆Ct control - sample)] / [2 reference^ (∆Ct control - sample)]

	Testis	Niere ♂	Leber ♂
	∆∆Ct	∆∆Ct	∆∆Ct
PAS1	1,000	1,000	1,000
PAS2	0,462	1,007	0,997
PAS3	0,083	1,009	0,981

	Testis	Niere ♂	Leber ♂
	+ SD	+ SD	+ SD
PAS1	0,085	0,102	0,106
PAS2	0,095	0,089	0,098
PAS3	0,086	0,092	0,065

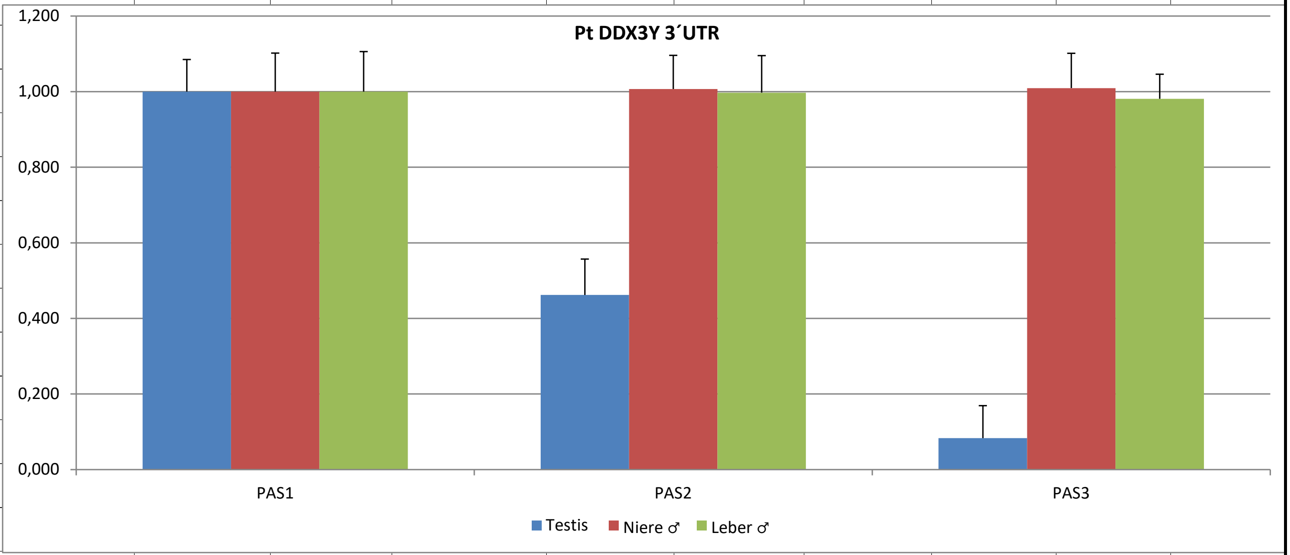

Quantitative Expression von DDX3Y-3´UTR-Transkripten in Gewebe von Mmul bezogen auf DDX3Y PAS1

25.06.2020

	PAS1	PAS2	PAS3
	Ct	Ct	Ct
Testis	19,600	20,254	24,310
Niere ♂	24,011	24,244	24,680
Leber ♂	23,610	23,617	23,597

ΔCt control - sample

	PAS1	PAS2	PAS3
	ΔCt	ΔCt	ΔCt
Testis	0,000	-0,654	-4,710
Niere ♂	0,000	-0,233	-0,669
Leber ♂	0,000	-0,008	0,013

Auswertung = [2 target^ (ΔCt control - sample)] / [2 reference^ (ΔCt control - sample)]

	Testis	Niere ♂	Leber ♂
	ΔΔCt	ΔΔCt	ΔΔCt
**PAS1**	1,000	1,000	1,000
**PAS2**	0,636	0,851	0,995
**PAS3**	0,038	0,629	1,009

	Testis	Niere ♂	Leber ♂
	+ SD	+ SD	+ SD
**PAS1**	0,078	0,089	0,065
**PAS2**	0,045	0,085	0,089
**PAS3**	0,098	0,075	0,102

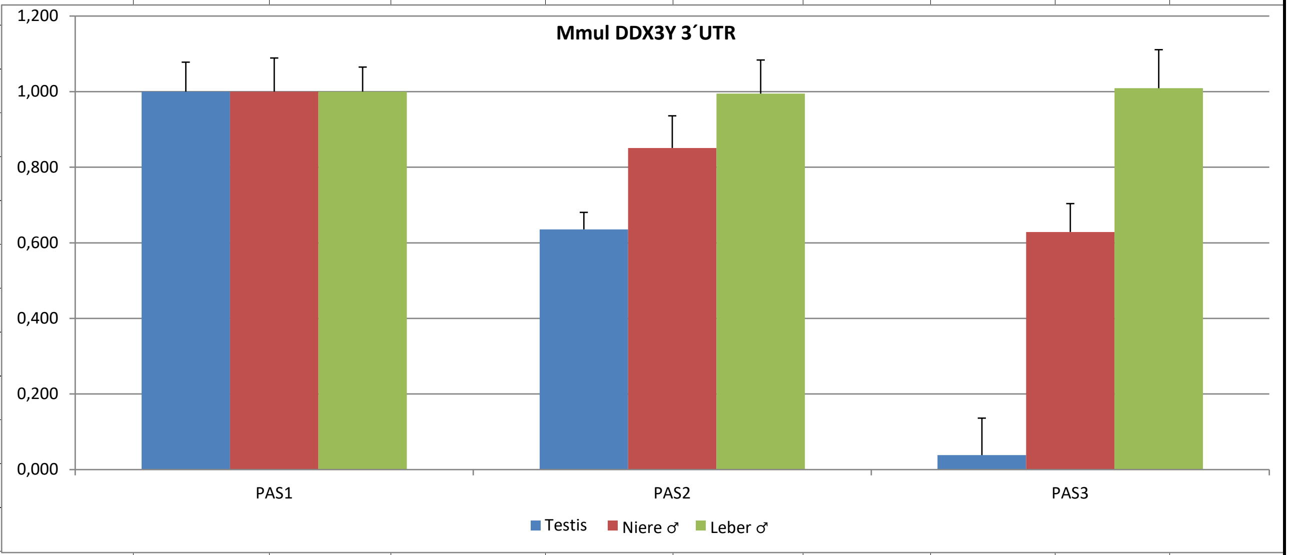

Quantitative Expression von DDX3Y-3´UTR-Transkripten in Gewebe von Cjc bezogen DDX3Y PAS1

25.06.2020

	PAS1	PAS2	PAS3
	Ct	Ct	Ct
Testis	28,625	28,642	28,808
Niere ♂	28,687	28,707	28,675
Leber ♂	29,065	28,997	29,081

∆Ct control - sample

	PAS1	PAS2	PAS3
	∆Ct	∆Ct	∆Ct
Testis	0,000	-0,017	-0,183
Niere ♂	0,000	-0,020	0,012
Leber ♂	0,000	0,068	-0,016

Auswertung = [2 target^ (∆Ct control - sample)] / [2 reference^ (∆Ct control - sample)]

	Testis	Niere ♂	Leber ♂
	∆∆Ct	∆∆Ct	∆∆Ct
**PAS1**	1,000	1,000	1,000
**PAS2**	0,988	0,986	1,048
**PAS3**	0,881	1,008	0,989

	Testis	Niere ♂	Leber ♂
	+ SD	+ SD	+ SD
**PAS1**	0,069	0,11	0,078
**PAS2**	0,065	0,089	0,103
**PAS3**	0,102	0,095	0,098

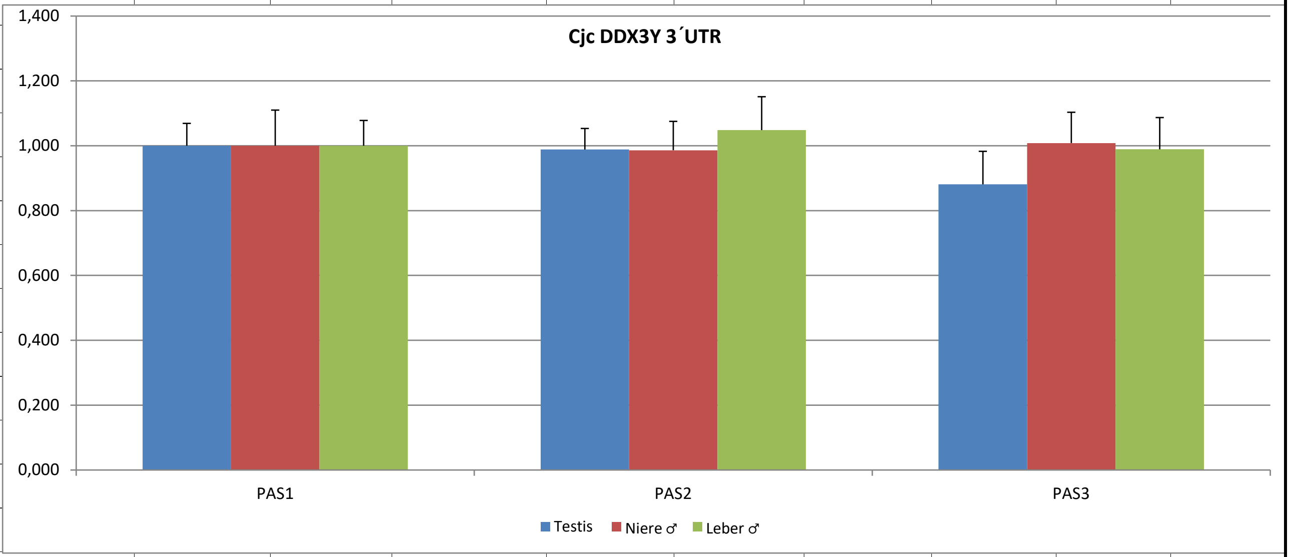

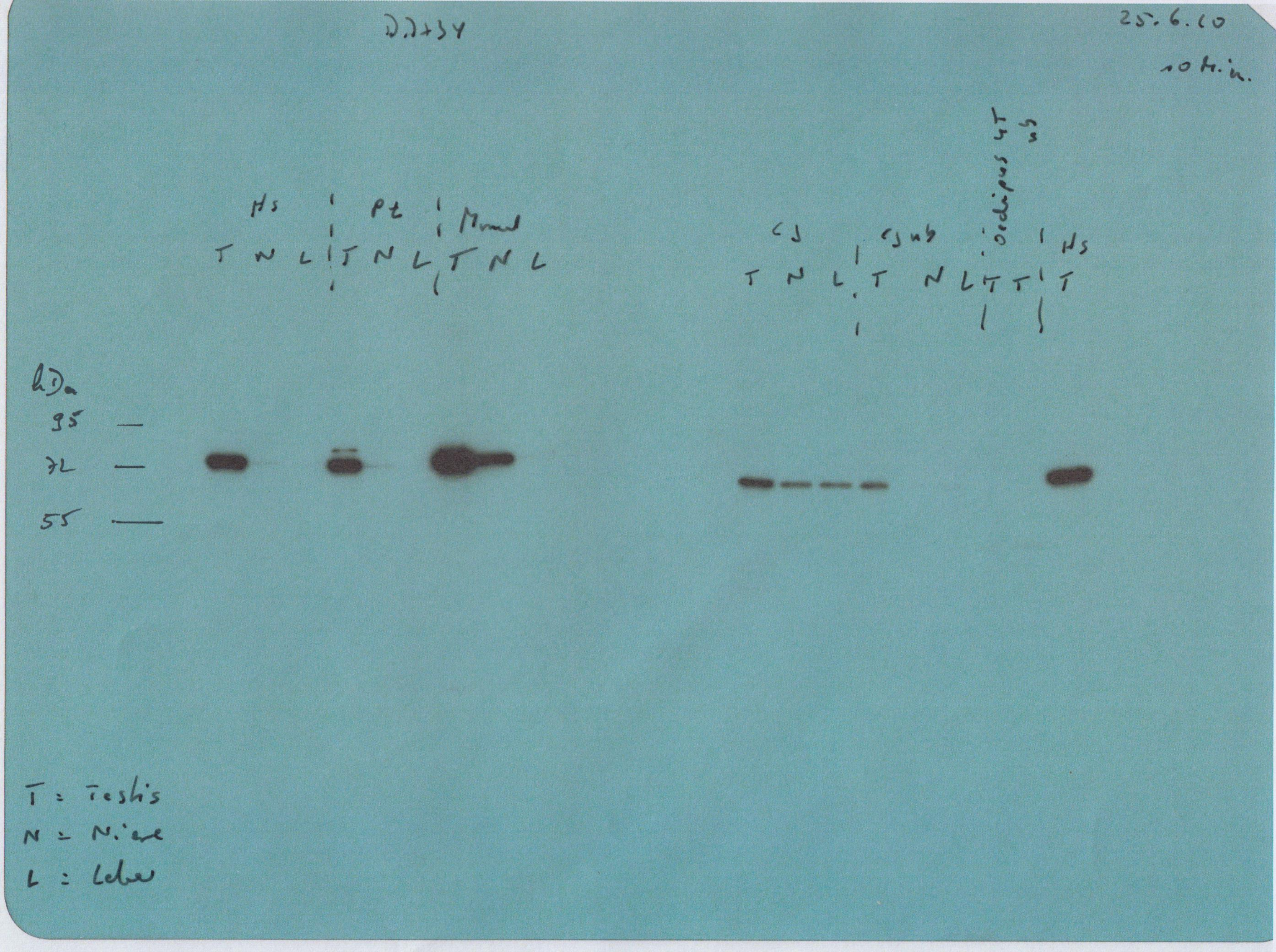
25.6.10
10 min.
Hs
Pt
T N L T N L T N L
Hs
T
kDa
95
72
55
T = Testis
N = Niere
L = Leber

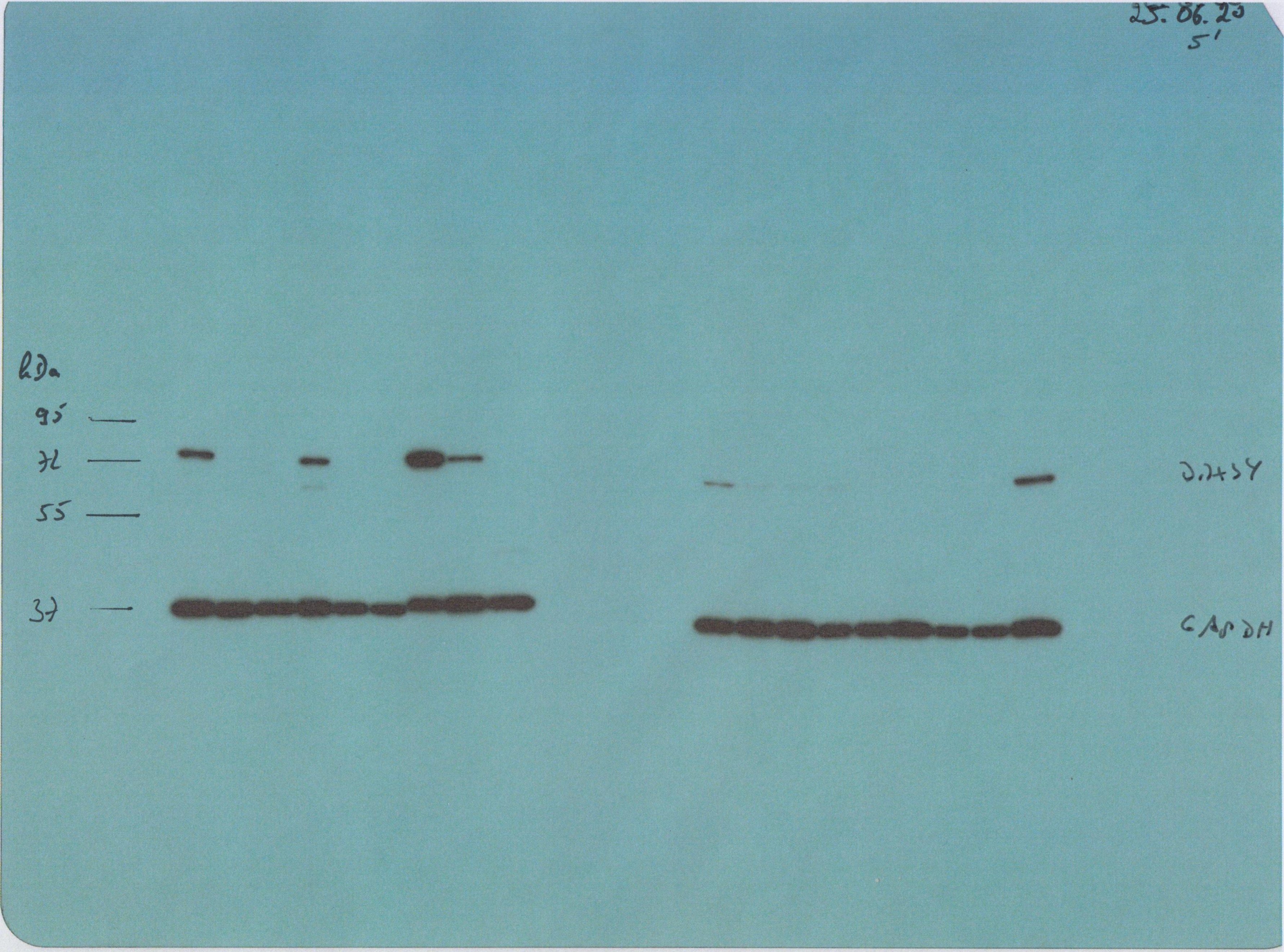
25.06.20
5'
kDa
95
72
55
37
GAPDH
